# Alertness fluctuations when performing a task modulate cortical evoked responses to transcranial magnetic stimulation

**DOI:** 10.1016/j.neuroimage.2020.117305

**Published:** 2020-12

**Authors:** Valdas Noreika, Marc R. Kamke, Andrés Canales-Johnson, Srivas Chennu, Tristan A. Bekinschtein, Jason B. Mattingley

**Affiliations:** a Queensland Brain Institute, University of Queensland, St Lucia, QLD 4072, Australia; b Cambridge Consciousness and Cognition Lab, Department of Psychology, University of Cambridge, Cambridge CB2 3EB, United Kingdom; c Department of Biological and Experimental Psychology, School of Biological and Chemical Sciences, Queen Mary University of London, Mile End Road, London E1 4NS, United Kingdom; d School of Computing, University of Kent, Medway, United Kingdom; e Department of Clinical Neurosciences, University of Cambridge, Cambridge, United Kingdom; f School of Psychology, University of Queensland, St Lucia, QLD 4072, Australia; g Canadian Institute for Advanced Research (CIFAR), Canada; h Vicerrectoría de Investigación y Posgrado, Universidad Católica del Maule, Talca, Chile

**Keywords:** Alertness, Electroencephalography (EEG), Motor evoked potentials (MEP), Transcranial magnetic stimulation (TMS), TMS evoked potentials (TEP)

## Abstract

Transcranial magnetic stimulation (TMS) has been widely used in human cognitive neuroscience to examine the causal role of distinct cortical areas in perceptual, cognitive and motor functions. However, it is widely acknowledged that the effects of focal cortical stimulation can vary substantially between participants and even from trial to trial within individuals. Recent work from resting state functional magnetic resonance imaging (fMRI) studies has suggested that spontaneous fluctuations in alertness over a testing session can modulate the neural dynamics of cortical processing, even when participants remain awake and responsive to the task at hand. Here we investigated the extent to which spontaneous fluctuations in alertness during wake-to-sleep transition can account for the variability in neurophysiological responses to TMS. We combined single-pulse TMS with neural recording via electroencephalography (EEG) to quantify changes in motor and cortical reactivity with fluctuating levels of alertness defined objectively on the basis of ongoing brain activity. We observed rapid, non-linear changes in TMS-evoked responses with decreasing levels of alertness, even while participants remained responsive in the behavioural task. Specifically, we found that the amplitude of motor evoked potentials peaked during periods of EEG flattening, whereas TMS-evoked potentials increased and remained stable during EEG flattening and the subsequent occurrence of theta ripples that indicate the onset of NREM stage 1 sleep. Our findings suggest a rapid and complex reorganization of active neural networks in response to spontaneous fluctuations of alertness over relatively short periods of behavioural testing during wake-to-sleep transition.

## 1 Introduction

Transcranial magnetic stimulation (TMS) is widely used for probing human brain function in health and disease (Dugué and VanRullen, 2017; Valero-Cabré et al., 2017; Ziemann, 2017). A number of neurophysiological indices of cortical TMS perturbation have been used to contrast experimental conditions of interest, including motor evoked potentials (MEPs) recorded from peripheral muscles (Barker et al., 1985; Bestmann and Krakauer, 2015) and TMS-evoked potentials (TEPs) which are thought to reflect the reactivity of underlying cortical circuits (Chung et al., 2015; Ilmoniemi et al., 1997). These and other outcome measures show varying sensitivity to different experimental manipulations, as well as confounding factors. Perhaps the largest within-participant variations in motor and cortical responses to TMS are observed when contrasting wakefulness and sleep. As healthy participants fall into slow wave sleep, MEP amplitude diminishes (Avesani et al., 2008; Bergmann et al., 2012; Hess et al., 1987; Grosse et al., 2002), whereas TEP amplitude increases in association with a breakdown of effective connectivity (Massimini et al., 2005, 2007). Likewise, sleep pressure has been shown to modulate TMS responses during normal waking in daytime hours (e.g., De Gennaro et al., 2007; Huber et al., 2013). It remains unknown, however, whether the effects of TMS on neural activity are influenced by spontaneous fluctuations in the level of alertness that may occur during a single experimental session.

Recent research has suggested that human participants can show widely varying levels of alertness throughout a testing session. For instance, Tagliazucchi and Laufs (2014) found that 30% of participants drifted into a drowsy state (N1 sleep) during resting-state functional magnetic imaging (fMRI) protocols after only three minutes. These periods of early N1 sleep during passive resting-state scans were accompanied by increased signal variance in sensory and motor cortices and altered cortico-cortical functional connectivity (Tagliazucchi and Laufs, 2014). Likewise, using an active decision-making task, De Gee et al. (2017) demonstrated that brainstem-controlled inter-trial fluctuations in phasic arousal are accompanied by changes in the involvement of prefrontal and parietal cortices in choice encoding. Further evidence for the contribution of fluctuating levels of alertness might come from studies of MEP amplitudes, which tend to be highly variable from trial to trial (Ellaway et al., 1998; Maeda et al., 2002). A significant portion of this variance is related to EEG oscillatory activity in a pre-TMS time window (Bergmann et al., 2019; Hussain et al., 2018; Mäki and Ilmoniemi, 2010; Madsen et al., 2019; Ogata et al., 2019; Sauseng et al., 2009; Thies et al., 2018; Zarkowski et al., 2006; Zrenner et al., 2018). In particular, trials with higher pre-stimulation alpha power tend to be associated with lower MEP amplitude (Ogata et al., 2019; Sauseng et al., 2009; Zarkowski et al., 2006), although null findings (Iscan et al., 2016) or a positive rather than negative correlation (Thies et al., 2018; Bergmann et al., 2019) have also been reported. The association between pre-stimulus alpha power and MEP amplitude is typically interpreted in terms of spontaneous fluctuation of regional sensorimotor mu-alpha rhythms (Bergmann et al., 2019; Hussain et al., 2018; Thies et al., 2018; Zrenner et al., 2018). Unfortunately, previous TMS investigations have not measured or controlled for changes in alertness in their participants, so it remains unknown whether fluctuations in alertness are systematically associated with changes in TMS-evoked neural activity.

Here we combined single-pulse TMS with concurrent EEG recording and a simple behavioural task to quantify changes in motor and cortical reactivity with fluctuating levels of alertness during wake-to-sleep transition, defined objectively on the basis of ongoing brain activity. We had four goals: (1) to estimate the latency and stability of fluctuations in alertness over the course of an active, single-pulse TMS session; (2) to test whether fluctuations in alertness modulate the occurrence and amplitude of MEPs; (3) to determine whether the amplitude of TEP responses within the first 50 ms after a TMS pulse changes across different levels of alertness; and (4) to assess whether inter-trial variance of MEP and TEP amplitudes is altered with decreases in alertness.

## 2 Materials and methods

### 2.1 Participants

Twenty participants (7 male; mean age 23.7 years: age range 21–33 years) took part in the study. All participants were screened for contraindications to TMS (Rossi et al., 2009), which included having no history of hearing impairment or injury, and no neurological or psychiatric disorders. All participants were right handed, as assessed using the Edinburgh Handedness Scale (Oldfield, 1971). The mean handedness index was 0.79 (SD=0.19; range 0.3–1). Potential participants were also screened with the Epworth Sleepiness Scale (ESS) (Johns, 1991). The mean ESS score was 9.4 (SD=4.3), which indicates that most of the participants had a slight to moderate chance of dozing off in a situation of prolonged inactivity. Notably, the average ESS score between 9 and 11 is typical for student samples (Kaur and Singh, 2017; Rodrigues et al., 2002; Yang et al., 2003; Zailinawati et al., 2009), which has been related to the pressures of studying and hectic lifestyles rather than clinical sleep problems (Hershner and Chervin, 2014).

The experimental protocol was approved by the Medical Research Ethics Committee of The University of Queensland (UQ), and the study was carried out in accordance with the Declaration of Helsinki. All participants gave informed, signed consent. Participants were recruited through an electronic volunteer database managed by UQ's School of Psychology. They received $30 for taking part in the study. There were no adverse reactions to TMS.

### 2.2 Electromyography (EMG)

**Fig. 1:**
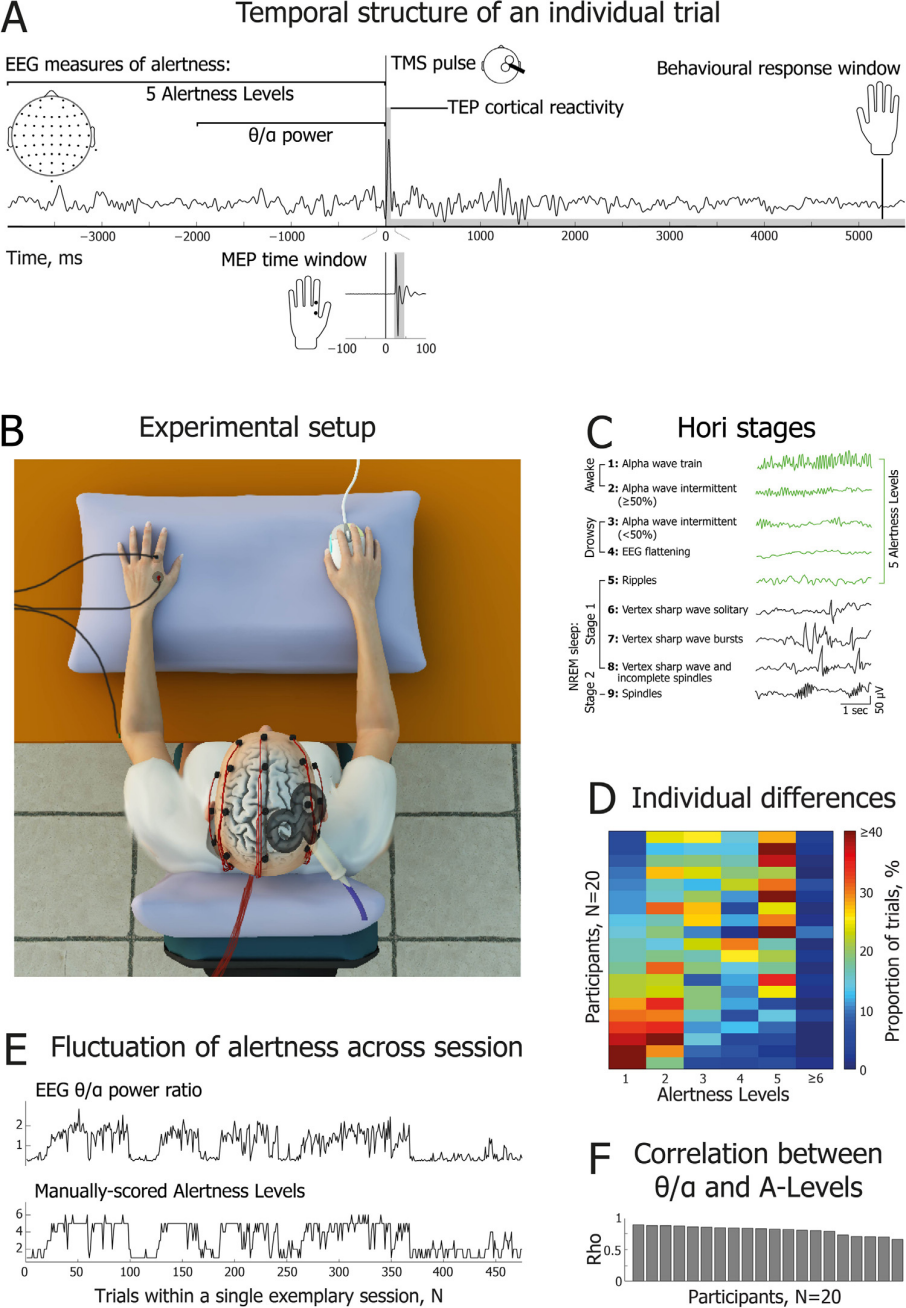
**| Experimental design and measurements of alertness.** (A) Temporal structure of an individual trial. Two EEG windows preceding single-pulse TMS were used to assess alertness: a 4 s window was used for manual scoring of Hori stages (5 Alertness Levels), and a 2 s window was used for automatic calculation of the theta (3–7 Hz) to alpha (8–12 Hz) spectral power ratio. Following each TMS pulse delivered over the right motor cortex, motor evoked potentials (MEPs) were recorded from the first dorsal interosseous (FDI) muscle of the left hand, and TMS-evoked potentials (TEPs) were recorded using high density EEG to characterize cortical reactivity. (B) Schematic of experimental set-up, showing EMG, EEG, TMS and response mouse in situ. (C) Brief definitions and EEG examples of 9 Hori stages of sleep onset, progressing from relaxed wakefulness (Hori Stage 1) to NREM Stage 2 sleep (Hori Stage 9) (modified with permission from Ogilvie (2001)). In the current study, Alertness Levels 1–5 (marked in green) correspond to Hori Stages 1–5. (D) Percentage of trials obtained within each Alertness Level, shown separately for the 20 participants. Datasets are sorted from the most alert participants (lower rows) to the drowsiest participants (upper rows). There were very few epochs of Alertness Level 6 or above. (E) Representative dataset for one participant, showing good agreement between the two EEG measures of alertness across the whole testing session. The upper subplot indicates *θ*/*α* ratio; the lower subplot shows fluctuations of Alertness Levels on the Hori scale. (F) Cross-validation of EEG measures of alertness: intra-individual correlations between θ/α power and the full range of Alertness Levels (Hori Stages 1–8) across single session trials. Bars represent intra-individual Spearman's rank order correlation coefficients for the 20 participants, sorted from the most to the least positive coefficients.

Surface EMG was recorded from the first dorsal interosseous (FDI) of the left and right hands using disposable 24 mm Ag–AgCl electrodes (Kendall H124SG by Covidien; MA, USA) (only left-hand EMG shown in Fig. 1(B)). The electrodes were placed in a belly-tendon montage with the reference over the proximal phalanx of the index finger and a common ground on the left elbow. Raw EMG signals were amplified (× 1000) and filtered (20–2000 Hz; 50 Hz notch filter) using a Digitimer NeuroLog system (Digitimer; Hertfordshire, UK). The data were digitised at 5000 Hz using a Power 1401 and Signal (v5) software (Cambridge Electronic Design; Cambridge, UK), and stored for offline analysis on a PC. Throughout the experiment EMG activity was monitored on-line using a digital oscilloscope with a high gain. Participants were prompted to relax if any unwanted muscle activity was observed.

### 2.3 Transcranial magnetic stimulation (TMS)

TMS was applied to the right primary motor cortex using a single monophasic pulse generated by a Magstim 200^2^ stimulator and a 70 mm figure-of-eight coil (#9925-00; The Magstim Company; Carmarthenshire, UK). The site for stimulation was the point on the scalp over the motor cortex that elicited the largest and most consistent amplitude MEPs from the left FDI. This stimulation ‘hotspot’ was found by placing the TMS coil tangentially on the scalp with the handle pointing posteriorly and laterally at ~45° to the sagittal plane, which induced a posterior-to-anterior current in the cortex. Stimulation commenced at an intensity that was assumed to be slightly suprathreshold for most individuals. Once the hotspot had been identified it was marked using an infrared neuro-navigation system (Visor 2 by ANT Neuro; Enschede, The Netherlands). A small piece of foam ~ 5 mm thick was then placed under the centre of the TMS coil so that it was not in physical contact with any EEG electrodes. The hotspot was re-marked and the location and orientation of the TMS coil were maintained throughout the testing session with the aid of the neuro-navigation system. Accuracy of coil position and handle orientation were kept within 5 mm and 5 degrees, respectively, but were typically within 3 mm and 3 degrees, as indicated in the Visor 2 panels. Resting motor threshold was determined using the relative frequency method with a criterion of ≥50 µV (peak-to-peak) MEP amplitude in at least five out of ten consecutive trials (Ikoma et al., 1996; Rossini et al., 1994; Samii et al., 1996). A two-down, one-up staircase was used, starting at a suprathreshold intensity. Mean motor threshold for the group was 53.1% (Range 34–74%) of maximal TMS output intensity. TMS was controlled manually during the localization of motor cortex and the estimation of motor threshold. During the main experiment, TMS was controlled via Matlab functions from the Rapid^2^ toolbox (Abrahamyan et al., 2011).

### 2.4 Electroencephalography (EEG)

Continuous EEG data were acquired using a 64 channel BrainAmp MR Plus amplifier, TMS BrainCap and Brain Vision Recorder (v1) software (Brain Products; Gilching, Germany). A high chloride abrasive electrolyte gel was used (Abralyt HiCl by Easycap; Herrsching, Germany), and electrode placement corresponded with the International 10–10 system. Data were sampled at 5 kHz with a bandpass filter of DC-1000 Hz and resolution of 0.5 µV (± 16.384 mV). Recordings were referenced online to the left mastoid, and electrode impedance was typically kept below 5 kΩ.

### 2.5 Experimental procedure

Participants were seated in a comfortable reclining chair that included head and leg support (see Fig. 1(B)). After placing the EMG and EEG electrodes, participants had their eyes blindfolded and the lights in the lab were dimmed. They were instructed to relax for a few minutes while estimation of individual resting motor threshold was performed. Participants’ hands were comfortably supported with pillows, and they wore earplugs throughout the experiment in order to reduce auditory stimulation. After threshold estimation the combined TMS-EEG experiment was carried out, and participants were reminded to stay relaxed and keep their eyes closed. They were also instructed to pay attention covertly to their left hand and to respond by clicking one of the two keys on a mouse held in their right hand if they felt a tactile sensation (left key), such as a twitch or a touch, or had no sensations (right key) in their left hand at the time of each TMS pulse (see Fig. 1(A)). In the present study, key presses were used to determine responsiveness. Participants were explicitly instructed that they were permitted to fall asleep should they wish to. If no responses were registered after 3–5 consecutive trials (i.e., failure to press a mouse button within 6 seconds of a TMS pulse), participants were gently awakened verbally and reminded to continue the task.

**Fig. 2:**
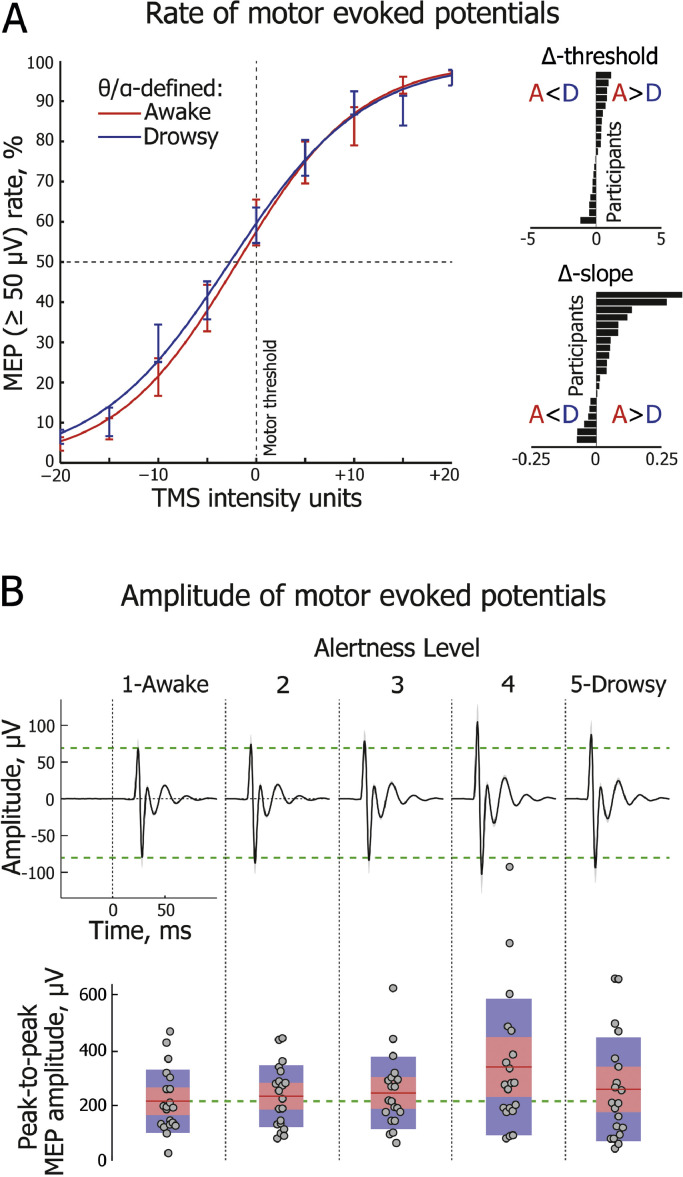
**| Motor evoked potentials (MEPs) shown across different levels of alertness.** (A) Group-averaged frequency of trials with MEPs above a threshold value of 50 μV across 9 TMS intensities, centred on individual motor thresholds (0%). Sigmoidal functions are fitted separately to the θ/α-defined awake (red) and drowsy (blue) conditions (error bars represent one standard error of mean, SEM). Insets on the right depict each participant's sigmoid threshold and slope difference (Awake minus Drowsy), with horizontal bars sorted in ascending order. Only responsive trials are included in the analysis shown in this and other subplots. Alertness states are distinguished here using the EEG θ/α measure taken from a 2000 ms window immediately prior to the TMS pulse. (B, upper panel) Group-level dynamics of MEPs (averaged across three TMS intensities centered on motor threshold) across Alertness Levels 1–5. Horizontal green dashed lines delineate peaks at 25 and 29 ms post-TMS (0 ms) for Alertness Level 1. (B, lower panel) Change in MEP peak-to-peak amplitude across Alertness Levels 1–5. Circles represent individual participants. For each Alertness Level, the red line depicts the group-level mean of peak-to-peak amplitude. The pink shaded region represents 1 standard deviation (SD), and the blue shaded region represents the 95% confidence interval of the mean.

During stimulation, nine TMS intensities centred on the individual resting motor threshold were used (−20%, −15%, −10%, −5%, 0%, +5%, +10%, +15%, +20%). Given that TMS stimulator output intensity is measured in whole numbers from 1 to 100, the calculated percentage from threshold intensity was rounded. This yielded slightly different sized steps from −20% to +20% for some individuals, and thus the actual TMS output intensity values were used when fitting sigmoidal functions at the single participant level. An a priori rationale for this design related to a secondary purpose of the project, which was to plot psychophysical functions of kinaesthetic awareness of hand movement, the results of which will be reported in a separate paper. For each individual, we aimed to deliver 520 trials of single pulse TMS, with an average inter-pulse interval of 9.5 s and a uniformly distributed random jitter of ±1000 ms. Thus, the inter-pulse interval lasted between 8.5 and 10.5 s. We incorporated a relatively long inter-pulse interval to facilitate the natural development of drowsiness, and to allow sufficient time for a return of tonic EMG activity to its baseline level. As our aim was to obtain the maximum number of evoked responses (MEPs and TEPs) around the TMS threshold intensity, the following number of trials was delivered at each TMS intensity: 40 trials (7.7% of a total) at each of the −20%, −15%, −10%, +10%, +15% and +20% intensities; 80 trials (15.4% of a total) at each of the −5% and +5% intensities; 120 trials (23.1% of a total) at 0% (i.e., at the individual resting motor threshold). These proportions of trials enabled us to maximize neural data around the motor threshold, while at the same time provided sufficient data to analyse sigmoidal fit as a function of TMS intensity (see Fig. 2(A)). Trial order was randomized throughout the experiment. TMS pulses were delivered in 8 blocks of 65 trials. In addition to the Rapid^2^ toolbox (Abrahamyan et al., 2011), the experiment was controlled using Matlab functions from Psychtoolbox-3 (Kleiner et al., 2007). Occasional technical difficulties meant that we could not complete all trials for every participant, or that we added an extra block of trials, yielding an average of 517.5 trials per participant (SD=35.84, Range=379–575).

One experimenter held the TMS coil, with the aid of the neuro-navigation system (Visor 2), and the other monitored ongoing EEG; these individuals switched their places after each block. An extended rest was provided after 4 blocks to allow participants a break from the task, to change the heated TMS coil, and to reduce the impedance of any EEG electrodes if required. Data collection lasted approximately 90 minutes. In an effort to reduce the potential impact of any circadian fluctuation in cortical excitability (Sale et al., 2007), all testing sessions commenced at 1.00 pm (a time at which participants were more likely to feel drowsy after having had their lunch).

### 2.6 Motor evoked potential (MEP) analyses

Peak-to-peak amplitudes of MEPs evoked by TMS pulses delivered over the right motor cortex were calculated for each trial within a 20–45 ms time window using Signal (v5) software (Cambridge Electronic Design; Cambridge, UK). Trials containing phasic muscle activity in the left FDI channel within 100 ms prior to a TMS pulse being delivered were discarded from the analyses.

We characterized modulations of MEPs as a function of alertness levels by fitting a sigmoid function to the proportion of trials that evoked MEPs (constrained from 0 to 1 on the *y* axis) across the 9 TMS intensities (−20%, −15%, −10%, −5%, 0%, +5%, +10%, +15%, +20), and then comparing threshold and slope measures in EEG-defined awake and drowsy trials separately for each participant (for definition of awake and drowsy trials, see Section 2.9). A 50 μV cut-off threshold in peak-to-peak amplitude (Ikoma et al., 1996; Rossini et al., 1994; Samii et al., 1996) was used to define the presence of an MEP. A sigmoid function was fitted to each individual participant's data using the following formula:

$$F=\frac{1}{1+e^{-\frac{x-\mu }{s}}}$$

where **F** is the MEP ratio (the proportion of trials with supra-threshold MEPs), **x** is the TMS intensity, **µ** is the threshold value (the TMS intensity at the inflection point), and **s** is inversely proportional to the slope at the threshold. The actual slope of the fitted sigmoid was calculated by fitting a straight line between a point 0.1 above the inflection point and a point 0.1 below it.

Importantly, in addition to spontaneous fluctuations in alertness, the spectral power of EEG pre-stimulus oscillations can reflect attentional sampling and/or sensory gating (Capotosto et al., 2009; Romei et al., 2008; van Dijk et al., 2008). We expected that an alertness-related effect would be spatially and temporally widespread and consistent, so we repeated the analysis of MEP threshold and slope by splitting the data between awake and drowsy trials separately for each EEG electrode, and in 20 equally sized pre-TMS time bins of 100 ms duration, from −2000 ms to 0 ms relative to the TMS pulse.

Furthermore, to assess dynamics of MEP peak-to-peak amplitude across Alertness Levels 1–5 (Hori et al., 1994; see Section 2.9), responsive trials with MEP amplitude at least twice as high as the range between minimum and maximum values in the −100 to 0 ms baseline window were averaged separately for each Alertness Level and each participant. In an effort to control for MEP variance as a function of TMS intensity, only three TMS intensities (−5%, 0%, +5%) around each individual's motor threshold were included in the group-level analysis of MEP amplitude changes across Alertness Levels 1–5.

### 2.7 EEG pre-processing and analysis: TMS-EEG reactivity

EEG data pre-processing was carried out using EEGlab toolbox for Matlab (Delorme and Makeig, 2004), with two separate pre-processing pipelines developed for the analysis of EEG reactivity to TMS and EEG spectral power before TMS pulses. Analysis of EEG reactivity to TMS pulses in the first 50 ms time window requires a perfect alignment of TMS markers in the EEG recording with the onset of the actual TMS coil discharge. Given that there was some delay and jittering between a TMS marker sent to the EEG system and the coil discharge itself (M=9.6 ms, SD=1.7 ms; see Fig. A.1, left side), EEG markers indicating TMS intensity were automatically adjusted to the time point of the actual TMS pulse. For this, raw EEG data were segmented ±200 ms around each TMS marker, and global field power (GFP) was calculated as a standard deviation of voltage across all electrodes, resulting in a single time waveform for each TMS marker. Each obtained waveform was baseline corrected to the −200 ms to −50 ms time window, and each time sample was transformed to its absolute value. The remaining time window of −49 ms to +200 ms was scanned, searching for the first time point at which a GFP value exceeded the maximal baseline GFP value by a factor of five, which indicated the onset of a TMS artefact. The TMS marker was then reallocated to this point in the continuous EEG recording (see Fig. A.1, right side).

The EEG data were processed following an ICA-based approach of TMS-EEG artefact cleaning (Rogasch et al., 2014). First, EEG data were segmented from −1000 ms to +1000 ms around the onset of the TMS artefact. Data were manually inspected and epochs containing excessive artefacts as well as epochs corresponding to noisy MEP recordings or pre-trial EEG segments used to assess alertness, were deleted (M=55.6 epochs, SD=30.37, Range=22–148). Next, the segments were baseline corrected to the mean of the interval from −500 ms to −100 ms time window. A straight line was then fitted to the data from −2 ms to 15 ms, thus deleting the initial TMS-EEG artefact, and the epochs were down-sampled to 1000 Hz. The most deviating EEG channels were then detected with the ‘spectopo’ function and the first round of independent component analysis (ICA) was performed excluding noisy channels (M=2.5 channels, SD=1.24, Range=1–6). After deleting on average 2.2 distinctive, early high-amplitude components (SD=1.47, Range=0–6) representing the exponential decay artefact (which probably results from a combination of the amplifier's step response, the induction of currents in the electrode leads, polarization of the electrode-electrolyte-skin interface, and cranial muscle response-related electrode movement (Herring et al., 2015; Rogasch et al., 2014, 2017) and is sometimes spread across many channels) a small number of additional noisy channels was identified (M=0.8 channels, SD=1.33, Range=0-5). EEG data were then filtered (1–80 Hz) and epoched from −400 ms to +600 ms around the onset of the TMS marker. The second round of ICA was carried out without the noisy channels identified in the two previous rounds (M=3.3 channels, SD=1.9, Range=1–9). Independent components reflecting the remaining TMS-EEG decay artefact, eye movements, auditory evoked potentials, 50 Hz line noise, and other sources of noise were deleted (M=20.75, SD=4.12, Range=15–29), after which bad channels were recalculated using spherical spline interpolation. The EEG segments were again baseline corrected (−100 ms to −3 ms), and manually inspected to delete a few remaining epochs that still contained a residual TMS artefact (M=2.7 epochs, SD=2.76, Range=0–9). After all data cleaning steps, on average 58.2 trials (11.2%) were discarded per single participant during EMG and EEG pre-processing (SD=30.82, Range=23–153), leaving on average 459.2 trials per participant (SD=36.44; Range=347–508 trials) available for the subsequent TEP and MEP analyses.

**Fig. 3:**
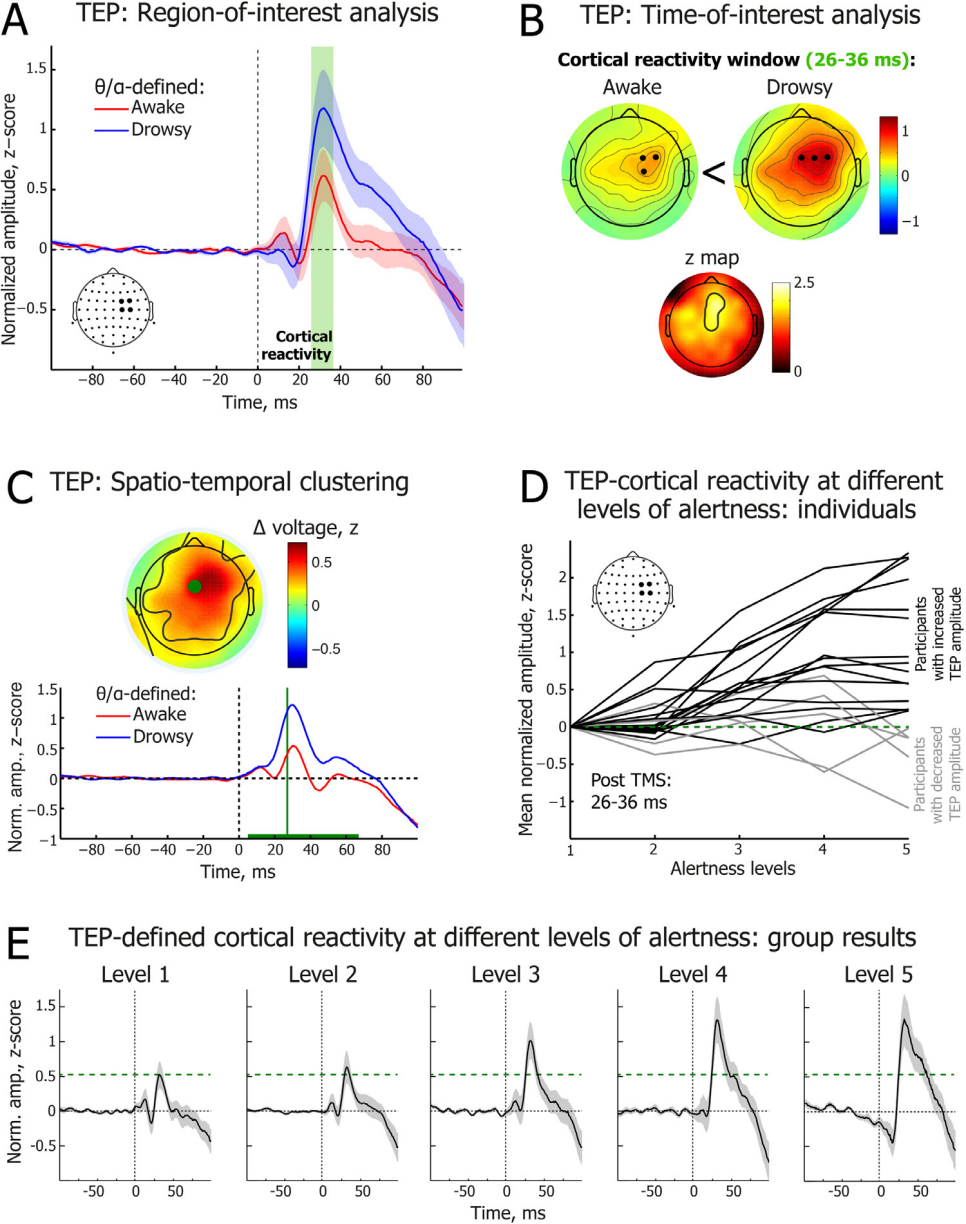
**| Transcranial magnetic stimulation-triggered cortical reactivity potentials (TEPs) across different levels of alertness.** (A) Time course of electroencephalography (EEG) potentials averaged over 4 EEG electrodes beneath the TMS coil in the θ/α-defined awake (red) and drowsy (blue) trials. Green shaded area highlights the cortical reactivity time window (26–36 ms). Only behaviourally responsive trials are included in the analysis shown in this and other subplots. 0 ms corresponds to the time of the TMS pulse. Red and blue shading depicts standard error of the mean (SEM). (B) Topographical distribution of the early TEP mean peak at 26–36 ms post-TMS pulse in the θ/α-defined awake (upper left) and drowsy (upper right) states. Black dots indicate locations of three EEG electrodes with the maximal amplitude in the map. Non-parametric *z* map (below) reveals region reliably different between awake and drowsy states. (C) 0–100 ms data-driven spatio-temporal clustering of EEG potentials post-TMS pulse between θ/α-defined awake (red) and drowsy (blue) states. TEP amplitude was significantly higher in drowsy trials in a 5-67 ms time window (cluster peak: 27 ms, *t* = 4884.47, *p* = 0.004). The green horizontal line depicts the time window of significant difference. The electrode with the largest difference between awake and drowsy states is marked as a green dot in the topographic voltage map, and its waveforms are plotted below. The black contours within the map show the electrodes with statistically reliable differences (cluster). The topographic voltage map is at the peak difference between awake and drowsy states. In addition to the P30 response, N45 and P60 TEP components are visible in this plot (Rogasch et al., 2014; Premoli et al., 2014). (D) Individual-level dynamics of TEP cortical reactivity peak amplitude across Alertness Levels 1-5 (TEP amplitude averaged over 26–36 ms across 4 electrodes beneath the TMS coil). Normalized amplitude is shown relative to Alertness Level 1 (green dashed line). Black lines represent participants with higher TEP amplitude at Ale-rtness Level 5 relative to Alertness Level 1 (*N*=15); grey lines represent participants with lower TEP amplitude at Alertness Level 5 relative to Alertness Level 1 (*N*=5). (E) Group-level dynamics of TEP waveforms across Alertness Levels 1–5 (TEPs averaged over 4 electrodes beneath the TMS coil). Horizontal green dashed line delineates TEP cortical reactivity peak at 31 ms post-TMS at Alertness Level 1.

To account for between-trial variance, the raw EEG signal from each individual trial was transformed to z-scores using the mean and standard deviation of the baseline period (−100 to −3 ms). Trials were then split into different levels of alertness. To assess changes in EEG reactivity to TMS perturbation as a function of alertness, the four electrodes immediately beneath the TMS coil (electrodes FC2, FC4, C2, C4) were chosen to contribute their voltage values to a region of interest (ROI) (see Fig. 3(A)), and these were then averaged across all TMS intensities within each participant. The group-level waveform was then plotted, revealing an early TEP peak at 31 ms post-TMS pulse. The data were then split between Alertness Levels and the mean amplitude (±5 ms) around the peak (26–36 ms) was calculated for each participant and each level of alertness (see Section 2.9).

ERP dynamics were additionally studied using data-driven spatio-temporal clustering analyses similar to what we have described previously (Chennu et al., 2013). Awake and drowsy trials were compared in the time windows of interest (15–100 ms) by averaging single-subject data and running group level clustering. Using modified functions from the FieldTrip toolbox (Maris and Oostenveld, 2007; Oostenveld et al., 2011), we compared corresponding spatio-temporal points, i.e. each electrode and each time sample, between awake and drowsy conditions, with a paired samples t-test. Although this step was parametric, FieldTrip uses a nonparametric clustering method (Bullmore et al., 1999) to address the multiple comparisons problem. The t values of adjacent spatio-temporal points whose p values were less than 0.05 were clustered together by summating their t values, and the largest such cluster was retained. A minimum of two neighbouring electrodes had to pass this threshold to form a cluster, with the neighbourhood defined as other electrodes within a 4 cm radius. This whole procedure – that is, calculation of t values at each spatio-temporal point followed by clustering of adjacent t values – was repeated 1000 times, with recombination and randomized resampling before each repetition. This Monte Carlo method generated a nonparametric estimate of the p value representing the statistical significance of the originally identified cluster. The cluster-level t value was calculated as the sum of the individual t values at the points within the cluster.

**Control analyses.** We conducted a number of control analyses to rule out the possibility that our specific approach to dealing with a variety of experimental artefacts unduly influenced the results. First, to determine whether transformation of the TEP data to z-scores might have influenced the results, all group level analyses were repeated in the voltage domain. Second, to rule out that removal of several auditory components following ICA might have influenced the results (M=1.95, SD=1.50, Range=0–5), the group level analyses were repeated with all auditory components retained. Third, to determine whether a different distribution of TMS intensities across awake and drowsy trials could have confounded the TEP results (see Table A.1), a number of trials at each TMS intensity was compared between θ/α-defined awake and drowsy conditions. In case of a mismatch trials were randomly drawn from a condition with the larger number to match another condition with the smaller number, and this was repeated separately for each participant. Once trial numbers were perfectly matched by TMS intensity between the states of alertness, the key contrast of TEP mean amplitude (26–36 ms) averaged across 4 electrodes beneath the TMS coil was repeated between θ/α-defined awake and drowsy conditions (see Section 2.9).

Finally, to determine whether variance in MEP amplitude could have confounded the TEP amplitude difference between awake and drowsy trials (e.g. through a sensory afferent signal re-entering the cortex), MEP peak-to-peak amplitude was statistically compared between θ/α-defined awake and drowsy conditions with an independent samples t test, separately for each participant. When a contrast yielded *p*<0.5, one trial with the largest amplitude was deleted from a condition with the larger mean amplitude and one trial with smallest amplitude was deleted from a condition with the smaller mean amplitude. Then, the awake and drowsy conditions were compared again, and the entire procedure was repeated until the obtained p value was ≥0.5. While initially MEP peak-to-peak amplitude was significantly higher in the drowsy condition for five participants and higher in the awake condition for one participant, no differences remained after awake and drowsy trials were matched by MEP amplitude (t(19): Mean=−0.12, Range=−0.65 to 0.60, p: Mean=0.67, Range=0.51–0.99), and the key contrast of TEP mean amplitude (26–36 ms) averaged across the 4 electrodes beneath the TMS coil, as well as across all electrodes, was repeated between θ/α-defined awake and drowsy conditions.

### 2.8 EEG pre-processing and analysis: pre-TMS spectral power

To calculate EEG spectral power before TMS, the recordings were downsampled to 250 Hz, and then epoched in −4000 ms to −12 ms time segments preceding each TMS pulse. The noisiest epochs were manually deleted, and the most deviant EEG channels were detected with the ‘spectopo’ function, before running the independent component analysis (ICA) for further removal of artefacts such as eye blinks and saccades, heartbeats, and muscle noise (deleted components: M=21.45, SD=3.71, Range=15–28). ICA was carried out on clean channels only, whereas the noisy channels were recalculated by spherical spline interpolation of surrounding channels after deleting ICA components with artefacts. Data were again manually inspected and several remaining noisy epochs were deleted.

The spectral power of EEG oscillations over the 4 s time interval immediately preceding each TMS pulse was computed using a Hilbert transform, set from 1.5 Hz to 48.5 Hz in steps of 1 Hz, using a finite impulse response (FIR) filter implemented in the EEGlab toolbox (Delorme and Makeig, 2004). Given that estimation of spectral power of slow oscillations can be difficult close to the edges of EEG segments (Cohen, 2014), and we were particularly interested in the spectral power just before each TMS pulse, a dummy copy of each EEG epoch was created by flipping the beginning and end of each pre-TMS epoch along the time axis, except of the last time point (−12 ms). The resulting “mirror image” data were then concatenated with the original pre-TMS data; that is, the time axis of the obtained 7.976 s EEG epochs extended from −4000 ms to −16 ms (original) to −12 ms (original) and then back from −16 ms to −4000 ms (mirror). In this manner, an abrupt discontinuity was avoided in the time window just before the TMS pulse, thus enabling a more stable estimate of spectral power. After Hilbert transformation, the “mirror” part of the EEG epoch was deleted, retaining the original pre-TMS window from −4000 ms to −12 ms. To reduce data size, EEG recordings were down-sampled to 250 Hz before running the Hilbert transform.

### 2.9 EEG measures of alertness

Two complementary EEG measures were used to assess participants’ level of alertness before each TMS pulse: (1) the Hori scoring system of sleep onset EEG (Hori et al., 1994), and (2) a ratio between EEG spectral power of pre-TMS theta and alpha oscillations, which we refer to here as the ‘θ/α’ measure of alertness (Bareham et al., 2014; Noreika et al., 2020).

The Hori system relies on visual scoring of 4 s segments of continuous EEG data (Hori et al., 1994). It consists of 9 stages reflecting a gradual progression from wakefulness to sleep, from Hori Stage 1 which refers to alpha-dominated relaxed wakefulness, to Hori Stage 9 which is defined by the occurrence of complete spindles coinciding with classic Stage 2 NREM sleep (see Fig. 1(C)). The Hori system has been used to map dynamic wake-sleep changes in ERPs (Nittono et al., 1999), EEG spectral power (Tanaka et al., 1997), reaction times, and the rate of subjective reports of being asleep (Hori et al., 1994). In the present study, Hori stages were visually assessed by an experienced sleep researcher (VN) who was blind to participants’ responsiveness and the TMS intensity on any trial. Given that the Hori system was developed using low-density EEG data, only 19 EEG channels of the standard 20-10 system were used for scoring purposes (Fp1, Fp2, F7, F3, Fz, F4, F8, C3, Cz, C4, T7, T8, P7, P3, Pz, P4, P8, O1, O2). EEG recordings were low pass filtered (20 Hz). Previous research has found that participants are typically unresponsive in Hori Stages 6 and above (Ogilvie, 2001), so our MEP and TEP analyses were restricted to Hori Stages 1-5, which we refer to here as ***Alertness Levels 1–5***.

Hori Stages 1 to 4 are marked by decreasing activity in the alpha range, and Hori Stages 4 to 8 are characterized by an increase in activity in the theta range (Hori et al., 1994). Thus, progression of drowsiness can be quantified by a ratio of the spectral power of the alpha and theta EEG frequency bands. Specifically, here drowsiness was quantified as a period of time with an increased θ/α ratio of spectral power (Bareham et al., 2014). To apply this measure, theta (4.5–7.5 Hz) and alpha (8.5–11.5 Hz) power was first averaged in time from −2000 ms to −12 ms, and the θ/α ratio was then calculated for each trial and electrode. Next, the θ/α ratio was averaged across all electrodes, resulting in a single “alertness” value per trial. Finally, trials were split into the most strongly “awake” (45%) and most strongly “drowsy” (45%) trials, excluding the 10% of trials that were intermediate between the two extremes. For the MEP and TEP analyses, unresponsive trials were deleted before carrying out the θ/α-split between awake and drowsy trials. However, all trials were used when the θ/α-split was carried out in order to compare the number of unresponsive trials between awake and drowsy states.

### 2.10 Convergence of the EEG measures of alertness

All participants completed the experimental task and reached the expected Alertness Level 5 or higher, marked by the occurrence and dominance of theta waves. At a group level, a comparable proportion of awake and drowsy trials were obtained as per the criteria defined above (Alertness Levels 1–2: M=45.17%, SD=19.92; Alertness Levels 4–5: M=35.68%, SD=16.44) (see Fig. 1(D)).

Thus, even though the Hori system provides absolute electrophysiological signatures of the depth of drowsiness, the θ/α ratio was used to identify equal proportions of awake and drowsy trials within each participant. Given that the θ/α measure is relative, there was a risk of mislabelling trials for some participants, as it would make a split between “awake” and “drowsy” trials even if all of them happened to be Alertness Level 1. Thus, to verify the use of θ/α data splits, we compared these two measures at an individual level and at the level of the group as a whole. First, we carried out correlation analyses between the two measures of alertness within each participant. Second, we compared correlation coefficients against zero to assess the consistency of association between the Hori and the θ/α measures. At an individual level, Hori stages 1–8 and θ/α scores were positively and significantly correlated for all 20 participants (individual rho ranged from 0.66 to 0.9). Group analysis confirmed a very strong association between these two electrophysiological measures of alertness (one sample *t* test: *t*(19)=51.99, *p*<0.000005), confirming that the θ/α ratio was well suited to assessing the level of alertness in the sample here (see Fig. 1(F)). A similar convergence between Hori and θ/α scores was observed when trials of Hori Stages 6–8 (M=2.23% of data, SD=2.74, Range=0–10.92) were excluded from correlations (individual rho ranged from 0.64 to 0.9; one sample *t* test: *t*(19)=50.75, *p*<0.000005).

### 2.11 Statistical analysis

Paired samples t tests were used to compare neural summary measures between θ/α-defined awake and drowsy states. Pooled variance was used to calculate Cohen's d, with 0.2 indicating a small effect size, 0.5 a medium effect size, and 0.8 a large effect size (Cohen, 1988). For a similar comparison of summary measures across Alertness Levels 1–5, a one-way repeated measures ANOVA was carried out with linear as well as non-linear contrasts. Huynh-Feldt correction was used when Mauchly's test indicated violation of the assumption of sphericity. Partial η^2^ was calculated as an effect size in ANOVA tests, with 0.01 indicating a small effect size, 0.06 a medium effect size, and 0.14 a large effect size (Cohen, 1988). A Shapiro-Wilk's test was used to assess normality of the distribution before running parametric tests. Square-root or log10 transforms were used to normalize skewed data. When transformations failed, non-parametric statistical tests were used, such as Wilcoxon's signed-ranks test instead of a paired samples t test, and Page's L trend test instead of a one-way repeated measures ANOVA for linear contrasts across Alertness Levels 1–5. Bonferroni–Holm multiplicity correction (Holm, 1979) of *p* values was carried out to account for the planned comparisons between baseline Alertness Level 1 and the other four Levels of Alertness. Non-parametric Spearman's rank order correlation tests were used to assess for an association between single-trial MEP and TEP responses across trials, separately for each participant, followed up by a one sample t test of rho values. Statistical analyses were carried out using Matlab and IBM SPSS (v25) software packages. Bayes Factors were approximated from the t value and sample size using JASP software (https://jasp-stats.org/).

## 3 Results

### 3.1 Fluctuation of alertness during single TMS sessions

On average, TMS sessions lasted for 92.5 min (SD=7, Range=73.5–104.3 min) including time spent switching TMS coils and allowing breaks for participants. During this period, all 20 participants reached Alertness Level 5 or higher (up to Level 8), reflecting deep drowsiness with a dominance of EEG theta ripples (see Fig. 1(C)). Notably, it took only 9.44 min on average for participants to reach Alertness Level 5 (SD=8.95, Range=2.25–33.35 min), indicating a rapid decrease of alertness despite the fact that eyes-closed participants were receiving TMS pulses and generating task-specific motor responses.

All participants ceased responding at some point during the testing session, after which they were either aroused spontaneously due to TMS, or they were prompted by an experimenter after 3 consecutive unresponsive trials. On average, 13.1% of trials were categorised as “unresponsive” (SD=9.7, Range=2.83–40.6), suggesting a notable impact of drowsiness on task performance. As expected, there were more unresponsive trials in the θ/α-defined drowsy trials (M=22.46%, SD=3.15) than in the awake trials (M=4.17%, SD=7.91; *t*(19)=6.84, *p*=0.000006, *d*=1.6). Likewise, the probability of unresponsive trials increased across Alertness Levels (Page's *L* trend test: *L*=1075, χ^2^=61.25, *p*=5E−15). Compared with Alertness Level 1 (Mean=0.82%, SD=1.18, Mdn=0), there were more unresponsive trials in Level 2 (Mean=2.5%, SD=4.55, Mdn=1.27; Wilcoxon signed-rank test: *Z*=2.02, *p*=0.044, *r*=-0.32), Level 3 (Mean=9.57%, SD=10.68, Mdn=5.44; Z=3.7, *p*=0.0004, *r*=-0.59), Level 4 (Mean=15.95%, SD=15.39, Mdn=12.87; Z=3.74, *p*=0.0005, *r*=-0.59) and Level 5 (Mean=35.03%, SD=19.75, Mdn=31.32; *Z*=3.92, *p*=0.0004, *r*=-0.62) trials, with all contrasts Bonferroni–Holm-corrected for multiple comparisons (Holm, 1979).

Alertness Levels and unresponsive trials tended to be spread across the testing session, i.e., participants tended to “oscillate” between awake and drowsy states (see Fig. 1(E) and Fig. A.2). Consequently, there was no systematic increase or decrease in Alertness Level within a session at the group level. Four participants showed a significant but weak positive correlation between Alertness Level and trial number (mean rho=0.21), 6 participants showed a significant negative association (mean rho=−0.27), and the remaining 10 participants showed no significant correlation between Alertness Level and trial number (mean rho=-0.008) (see Fig. A.2). These results suggest that a given participant's level of alertness cannot be assumed to decrease continuously over a testing session. Only concurrent EEG measures can definitively determine a participant's moment-to-moment level of alertness.

### 3.2 Fluctuating levels of alertness modulate MEPs

We first assessed corticospinal excitability as a function of alertness and TMS intensity. To this end we calculated the proportion of trials with MEP peak-to-peak amplitude above 50 μV for each of the 9 TMS intensities, separately for the θ/α-defined awake and drowsy trials. A sigmoid function was then fitted across alertness conditions for each participant. The slope of the MEP sigmoid was slightly but significantly shallower in drowsy compared with awake trials (Wilcoxon signed-rank test: *z*-score=2.02, *p*=0.044, *r*=0.32), suggesting mildly increased noise and instability in corticospinal processing (see Fig. 2(A); individual participant results are shown in Fig. A.3). At the group level, the MEP slope difference between awake and drowsy trials was mainly driven by more the frequent occurrence of MEPs at subthreshold intensities (see Fig. 2(A)). At the individual level, some participants also showed an increase or decrease in MEPs at suprathreshold intensities (see Fig. A.3). Contrary to the slope findings, the MEP sigmoid threshold did not differ between awake and drowsy trials (*t*(19)=1.31, *p*=0.21, *d*=0.13, Bayes Factor in favour of the null=2.04).

We considered whether the observed difference in MEP slopes was specifically related to alertness, as the amplitude of pre-stimulus alpha oscillations has also been implicated in fluctuations in attention and sensory gating. For instance, it is possible that EEG markers of alertness could be confounded by a rapid co-linear fluctuation in attention. However, in these cases, EEG alpha effects are typically evident only within a relatively short pre-stimulus time period of a few hundred milliseconds (Romei et al., 2008), and are restricted to sensory or fronto-parietal regions (Capotosto et al., 2009; van Dijk et al., 2008). Contrary to this, the difference observed here in MEP sigmoid slope as a function of EEG θ/α power was temporally and spatially widespread (Fig. A.4), consistent with slow and widely distributed changes in alertness. Interestingly, the strength of association between theta/alpha ratio and MEP slope seemed to fluctuate at a 1 Hz frequency, with a significant peak observed 600–100 ms before TMS, and another peak at 1600–1200 ms before TMS (see Fig. A.4(A)). At the other pre-TMS time bins, the association was not significant but showed a trend in the same direction, i.e. a shallower MEP slope for deeper levels of drowsiness. Thus, we conclude that single-trial EEG θ/α power indeed reflected an instantaneous level of alertness rather than spontaneous fluctuations of attention linked to the phase of alpha oscillations.

We next compared MEP peak-to-peak amplitudes between Alertness Level 1, reflecting relaxed wakefulness, and Alertness Levels 2–5, reflecting increasing levels of drowsiness. As shown in Fig. 2(B), there was a significant increase in MEP amplitude between Alertness Levels 1 and 4 (*t*(19)=3.5, *p*=0.0096, *d*=0.64; Bonferroni–Holm-corrected (Holm, 1979)), as well as an intermediate stepped increase across Alertness Levels 2 (*d*=0.15; n.s.) and 3 (*d*=0.23; n.s.) and a subsequent decrease at Alertness Level 5 (*d*=0.27; n.s.). A linear trend of increasing MEP amplitude was observed across Alertness Levels 1-4 (*F*(1,19)=11.55, *p*=0.003, partial *η*^2^=0.38), but this was no longer significant when Level 5 was also included (*F*(1,19)=2.11, *p*=0.165, partial *η*^2^=0.1). These findings indicate a reliably non-linear reorganization of corticospinal excitability at a time when drowsy participants are still conscious and responsive. The most noticeable change in dynamics occurred with the disappearance of alpha waves at Alertness Level 4, at a point where there was EEG flattening just before the first occurrence of EEG theta-range ripples, despite the fact that participants were still responding behaviourally in the task. These observations suggest a much earlier modulation of corticospinal excitability in the initial moments of drowsiness than has been reported previously in studies of MEP changes with sleep deprivation or during NREM sleep (De Gennaro et al., 2007; Grosse et al., 2002; Manganotti et al., 2004).

At a single participant level, however, variance of MEP peak-to-peak amplitude explained by EEG θ/α ratio was negligible (for single-participant linear regression models, see Table A.2). While TMS intensity (−20% to +20%) explained on average 23.5% of single-trial MEP amplitude variance (SD=10.3, Range=0.5–42.3, significant in 19/20 participants), θ/α ratio alone explained on average only 1.8% of MEP variance (SD=2.8, Range=0–10.6, significant in 7/20 participants). Even though the additional variance explained by the θ/α ratio, over and above that of TMS intensity, was significant in 9/20 participants, it did not substantially increased the variance explained by TMS intensity alone (M=25%, SD=10.7, Range=0.5–42.4).

### 3.3 Fluctuating alertness modulates TMS-evoked potential (TEP) amplitude

We next assessed post-TMS cortical reactivity measured as TMS-evoked potentials (TEPs) within the first 40 ms after each pulse. Early TEP amplitude is known to increase in response to homeostatic sleep pressure (Huber et al., 2013) and during NREM sleep (Massimini et al., 2005), likely reflecting a combination of synaptic strengthening, changes in neuromodulation, and impaired inhibition (Huber et al., 2013). We hypothesized that, as with MEP amplitude, TEPs should be affected by the level of alertness in drowsy but responsive participants.

Comparing TEP amplitudes between θ/α-defined awake and drowsy trials revealed a significant increase in cortical reactivity in drowsy trials at the ROI electrodes in a time window from 26 to 36 ms after the TMS pulse (*t*(19)=4.02, *p*=0.00074, *d*=0.49) (see Fig. 3(A)). This pattern was evident in 18/20 participants (see Fig. A.5). Essentially the same increase in TEP amplitude was observed when θ/α-defined awake and drowsy trials were matched across TMS intensity conditions (see Fig. A.6) and MEP peak-to-peak amplitude (see Fig. A.7).

While TEP peaked over the right motor region, directly beneath the TMS coil, in both states of alertness, the peak difference between awake and drowsy states was fronto-central (see Fig. 3(B)). Consistent peak time and location were identified using spatio-temporal clustering of TEP differences between awake and drowsy trials (see Fig. 3(C)). While the observed difference between awake and drowsy trials spread fronto-centrally (see Fig. 3(B)–(C)), its occurrence is unlikely to reflect an auditory ERP as the TEP peak latency (27 ms) occurred well before the known onset of the auditory P50 potential (Pratt et al., 2008). Furthermore, we removed identifiable auditory components during ICA cleaning of the EEG signal, as outlined in the Materials and Methods (Section 2.7).

We further compared TEP amplitudes at the ROI site of stimulation across Alertness Levels 1–5. As hypothesized, TEP amplitude increased as participants became drowsier (Page's *L* trend test: *L*=1007, *χ*^2^=22.9, *p*=0.0000017) (Fig 3(D)–(E)). Planned comparisons between Alertness Level 1, which was treated as a baseline condition, and each subsequent Level revealed a significant increase in TEP amplitude between Alertness Levels 1 and 3 (*t*(19)=4.54, *p*=0.00088, *d*=0.51), 1 and 4 (*t*(19)=4.38, *p*=0.00099, d=0.68), and 1 and 5 (*t*(19)=3.43, *p*=0.0056, d=0.6), but not between Levels 1 and 2 (*t*(19)=1.54, *p*=0.14, *d*=0.12), with all contrasts Bonferroni–Holm-corrected for multiple comparisons (Holm, 1979). These findings provide the first direct evidence for an inverse association between cortical reactivity and alertness, suggesting that sleep-related changes in neural activity may intrude early in the transition between wakefulness and sleep, while participants are still able to respond in an ongoing behavioural task. Strikingly, the TEP effects emerged at a relatively early Alertness Level 3, before the appearance of drowsiness ripples or early slow waves (Hori et al., 1994).

Essentially the same increase in TEP amplitudes was evident in the θ/α-defined drowsy condition and across Alertness Levels 1–5 when all the aforementioned TEP analyses were repeated in the voltage domain (see Fig. A.8). Likewise, we observed the same effect when all the TEP analyses were repeated with auditory independent components retained in the EEG data, both in the voltage domain (see Fig. A.9) and using z-scores (see Fig. A.10).

At a single participant level, variance in TEP mean amplitude explained by EEG θ/α ratio was on average 5% (SD=4.9, Range=0–13.7, significant in 14/20 participants), which was comparable to the TEP variance explained by TMS intensity (*M*=4.9%, SD=7, Range=0.2–23.9, significant in 13/20 participants). Additional variance explained by the θ/α ratio, over and above TMS intensity, was significant in 14/20 participants. Thus, there was an additive effect of TEP variance explained by both predictors (*M*=9.5%, SD=9.6, Range=0.4–30.9). For single-participant linear regression models of TEP amplitude, see Table A.3.

### 3.4 Single-trial MEP and TEP variability across different levels of alertness

Having examined group-level changes in MEP and TEP amplitudes with spontaneous fluctuations in alertness, we next carried out single-trial analyses of TMS-evoked response variability, separately for each Alertness Level. Response variability was quantified as the intra-individual standard deviation (intraSD) of the TMS-evoked response amplitude, calculated separately for each participant and Alertness Level. To reduce the impact of uneven trial numbers across the five levels of Alertness, trials were randomly sampled for each participant to match an Alertness level with the smallest number of trials. Given that MEP amplitudes are strongly skewed, intraSD was calculated over log-transformed MEP amplitudes, and the obtained intraSD values were subjected to a group-level analysis of trend across the five levels of Alertness.

**Fig. 4:**
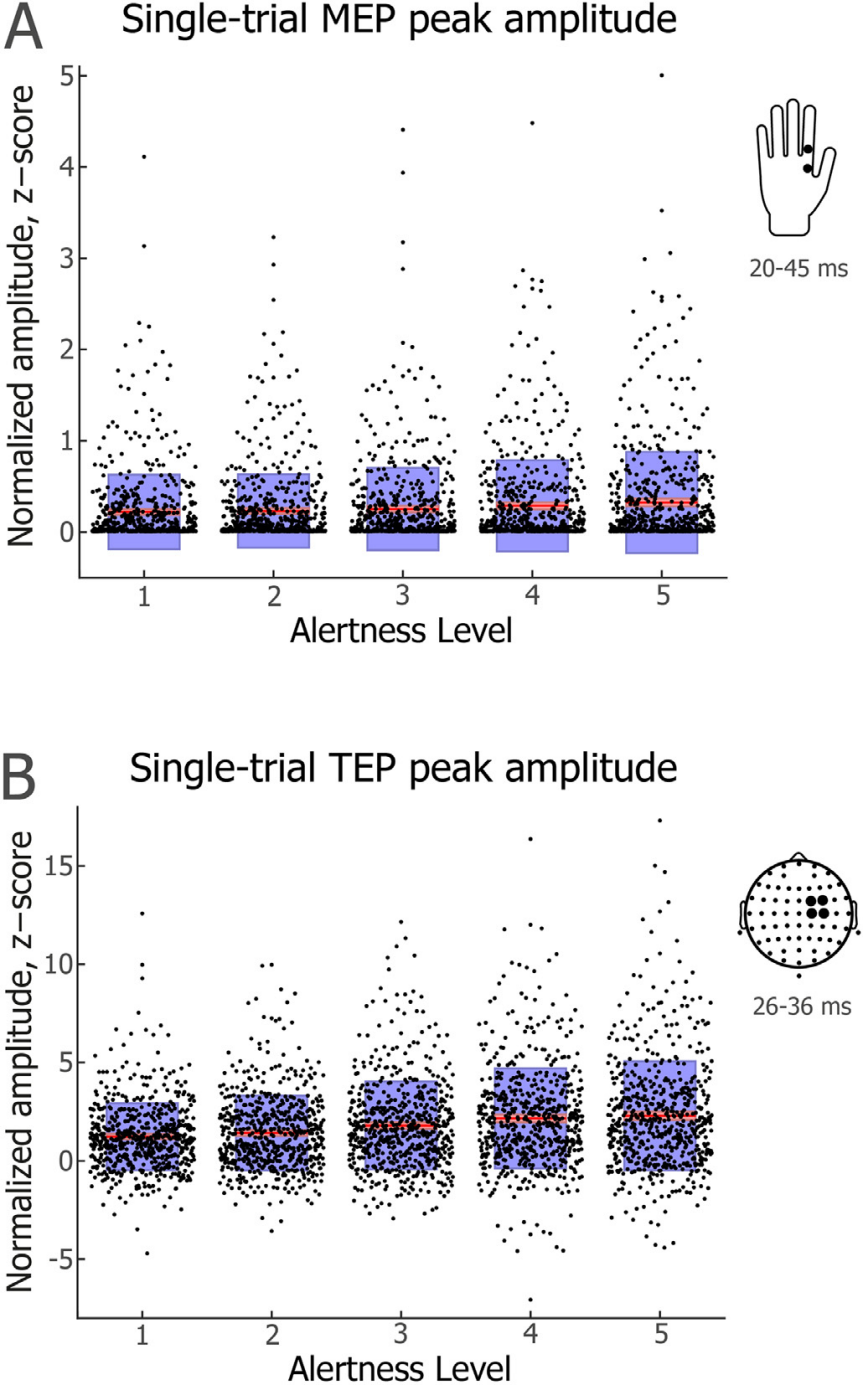
**| Single-trial MEP and TEP amplitudes across different levels of alertness.** (A) MEP peak-to-peak amplitude. (B) TEP peak amplitude. Jittered dots represent individual trials across participants (*N*=613 per condition). For each Alertness Level, the red line depicts mean amplitude. Pink shading represents 1 standard deviation (SD), which was very small and is thus difficult to discern in the figure. Blue shading represents the 95% confidence intervals for the mean. Insets on the right indicate locations of hand and scalp electrodes and time windows used to detect peak amplitude values.

The intraSD of single-trial MEP amplitude did not vary consistently across Alertness Levels 1–5 (linear contrast ANOVA: *F*(1,19)=3.34, *p*=0.083, partial *η*^2^=0.15; see Fig. 4(A)). Likewise, there was no significant difference between Level 1 and any other Alertness Level (lowest *p*=0.24).

By contrast, the intraSD of single-trial TEP amplitude increased significantly across Alertness Levels 1–5 (Page's *L* trend test: *L*=1024, *χ*^2^=30.75, *p*=3E-08; see Fig. 4(B)). Relative to Alertness Level 1 (*M*=1.25, Mdn=1.28), TEP amplitude variability was higher at Level 2 (M=1.57, Mdn=1.51, Wilcoxon signed-rank test: *Z*=2.99, p=0.0028, *r*=−0.47), Level 3 (M=1.77, Mdn=1.70; *Z*=3.81, *p*=0.0006, *r*=-0.60), Level 4 (*M*=2.01, Mdn=2.03; *Z*=3.62, p=0.007, *r*=−0.57) and Level 5 (M=1.93, Mdn=1.75; *Z*=3.62, *p*=0.007, *r*=-0.57), with p values Bonferroni–Holm-corrected for multiple comparisons (Holm, 1979).

### 3.5 Relationship between single-trial MEP and TEP amplitudes across levels of alertness

In a final step, we asked whether MEP and TEP amplitudes were correlated at a participant level, in line with previous studies (Fecchio et al., 2017; Petrichella et al. 2017), and whether any such association was modulated by Alertness Levels. We first carried out intra-individual Spearman's rank order correlations between MEP and TEP amplitudes across all TMS intensities, separately for each participant and Alertness Level. We then tested the group-level distribution of the obtained correlation coefficients by running one sample t tests, separately for each Alertness Level (see Fig A.11). While uncorrected probability values pointed to a significant association at each Alertness Level, only Level 3 showed a significant correlation between MEP and TEP amplitudes after Bonferroni–Holm correction of p values (Holm, 1979): Level 1 (*t*(19)=2.67, p_UNCORRECTED_=0.015, p_CORRECTED_=n.s.), Level 2 (*t*(19)=2.57, p_UNCORRECTED_=0.019, p_CORRECTED_=n.s.), Level 3 (*t*(19)=4.25, p_UNCORRECTED_=0.00043, p_CORRECTED_=0.0021), Level 4 (*t*(19)=2.48, p_UNCORRECTED_=0.023, p_CORRECTED_=n.s.), and Level 5 (*t*(19)=2.14, p_UNCORRECTED_=0.046, p_CORRECTED_=n.s.). There was no consistent change in the association between MEP and TEP amplitudes across Alertness Levels 1–5 (linear contrast ANOVA: *F*(1,19)=1.50, *p*=0.24, partial *η*^2^=0.073).

## 4 Discussion

Most studies that use TMS to investigate perceptual, cognitive and motor function in human participants do not consider the possibility that fluctuating levels of alertness across a single daytime testing session might lead to measurable changes in the associated patterns of brain activity. Here we used single-pulse TMS delivered over the right motor cortex while simultaneously measuring MEPs and TEPs across different, objectively defined levels of alertness while eyes-closed participants engaged in a simple tactile perception task. Participants exhibited fluctuating levels of alertness across the testing session, as indexed by continuous EEG recordings, but continued to respond behaviourally even in relatively deep states of drowsiness corresponding to the onset of NREM Stage 1 sleep (Alertness Level 5). Strikingly, both motor evoked responses and TMS-evoked cortical reactivity were altered across different levels of alertness. Specifically, we found that MEP amplitudes peaked during EEG flattening (Alertness Level 4), whereas TEP cortical reactivity increased earlier and remained stable across Alertness Levels 4 and 5. Our findings highlight that a proportion of inter-trial variability in neurophysiological responses to TMS, in particular TEP amplitude, can potentially be attributed to spontaneous fluctuations in alertness during wake-to-sleep transition.

Inter-trial and inter-subject variability in MEP amplitude is a well-known source of data variance in TMS experiments (Kiers et al., 1993; Ellaway et al., 1998; Rösler et al., 2008; Schutter et al., 2011; Sommer et al., 2002), and it has been suggested that 30 or more trials are required to provide a reliable estimate of MEP amplitude (Goldsworthy et al., 2016). The non-stationarity of MEP amplitudes has been attributed to a number of factors, including pre-stimulus voluntary muscle contraction (Kiers et al., 1993), variation in the number of recruited alpha-motor neurons (Rösler et al., 2008), variation in the synchronization of motor neuron discharges (Rösler et al., 2008) and functional hemispheric asymmetries (Schutter et al., 2011). Furthermore, a series of studies found that the amplitude or phase of pre-TMS EEG oscillations can predict MEP amplitude, including the alpha (Bergmann et al., 2019; Hussain et al., 2018; Ogata et al., 2019; Sauseng et al., 2009; Schulz et al., 2013; Thies et al., 2018; Zarkowski et al., 2006), beta (Mäki and Ilmoniemi, 2010; Keil et al., 2014; Ogata et al., 2019; Schulz et al., 2013; Zrenner et al., 2018) and gamma (Zarkowski et al., 2006) frequency bands.

In most recent MEP studies, a significant association between pre-TMS alpha oscillations and MEP amplitude was linked to the sensorimotor mu-rhythm, which shows spatially local effects confined to one or two pre-TMS cycles of mu (Bergmann et al., 2019; Hussain et al., 2018; Madsen et al., 2019; Ogata et al., 2019; Thies et al., 2018; Zrenner et al., 2018). For instance, a recent study by Ogata et al. (2019) found a significant positive association between pre-TMS alpha and MEP amplitude in an eyes-open condition, but not in an eyes-closed condition. While this could be interpreted in terms of an alertness difference between eyes-open and eyes-closed conditions, the reported alpha effect was typically restricted to the −250 ms to 0 ms time window and the motor cortex stimulation site, pointing to the sensorimotor mu-rhythm. Contrary to this, our pre-TMS EEG effects were spread both in the pre-stimulation time and electrode space (see Fig. A.4), indicating that slow fluctuations in alertness rather than sensorimotor mu-rhythm was driving the observed MEP changes. Thus, our study extends previous research by demonstrating that changing levels of alertness could be an important factor in brain state-modulation of corticospinal excitability. A related effect was reported by Zarkowski et al. (2006), who demonstrated that MEP amplitude is negatively correlated with pre-TMS alpha power (10–13 Hz) and positively correlated with pre-TMS gamma power (30–60 Hz), with an alpha/gamma ratio being the strongest predictor of MEP amplitude. While a theta/alpha ratio can index the level of alertness in eyes-closed experiments, such as in the present study, the alertness-indexing frequencies are shifted upward in eyes-open paradigms (Eoh et al., 2005; Kaida et al., 2006; Zhao et al., 2012). It is therefore likely that the results of Zarkowski et al. (2006), similarly to our study, were influenced by changing levels of alertness in the participant sample.

Linear regression analysis revealed that unique variance of single-trial MEP peak-to-peak amplitude explained by EEG θ/α ratio was on average just 2%. However, this could be due to the non-linear association between MEP amplitude and alertness, i.e. the increase of MEP amplitude at Alertness Level 4. Our finding of non-linear changes in MEP amplitude with decreasing levels of alertness might also explain previous contradictory findings regarding sleep deprivation effects on MEP amplitude. While several studies have reported an increase in corticospinal motor threshold following sleep deprivation (Manganotti et al., 2001; De Gennaro et al., 2007), other studies have failed to find any such effect (Civardi et al., 2001; Manganotti et al., 2006). Arguably, due to individual differences in instantaneous drowsiness levels, and potentially different times of day and durations of testing, the dominant level of alertness varied between these studies, confounding their comparison. For instance, datasets with a relatively high proportion of trials obtained during Alertness Level 4 would likely indicate higher MEP amplitude compared with other datasets. Unfortunately, a fine-grained measurement of alertness is seldom undertaken in MEP studies, even when EEG is recorded, e.g., “sleepiness” or NREM Stage 1 sleep are usually treated as a uniform state (Manganotti et al., 2004), even though a more detailed analysis can reveal at least 4 micro-states within N1 sleep (Hori et al., 1994; see Fig. 1(C)).

Regarding the modulation of TEPs with sleepiness, Huber et al. (2013) observed an increase in TEP amplitude as a function of prolonged wakefulness as well as following sleep deprivation. Contrary to our results, however, they found no association between TEP amplitude and short-lasting episodes of drowsiness. This discrepancy could be attributable to the fact that Huber et al. (2013) followed a behavioural definition of drowsiness (specifically, performance in a visuomotor tracking task), kept behavioural sessions much shorter (2–3 min) and instructed their participants to keep their eyes open. Furthermore, while the sessions in Huber et al. (2013) were likely too short for drowsiness to develop following the baseline night, it is feasible that TEP amplitude increase following sleep deprivation was at ceiling, and participants’ instantaneous level of alertness could not modulate it any further. Contrary to this, we used fine-grained EEG measures of alertness that could be quantified independently of fluctuations in behaviour, our testing sessions were longer, and participants were instructed to close their eyes, which facilitated spontaneous fluctuations in alertness. In another recent study, TEP amplitude was found to depend on the interaction between sleep pressure and phase of the circadian cycle rather than sleep homeostasis alone (Ly et al., 2016). Furthermore, the same study found that an increase in TEP amplitude was associated with an increase in EEG theta power across 29 h of sustained wakefulness. Unfortunately, the authors did not report whether such an association held over a shorter period of time (e.g., 45–90 min), as would be the duration of experiments in typical TMS studies. Our study thus complements and extends these previous reports by demonstrating a much more rapid increase of TEP amplitude in response to spontaneous fluctuations of alertness.

The TEP amplitude increase we observed during wake-to-sleep transition is reminiscent of the TEP amplitude increase previously found for reduced levels of alertness, such as during NREM sleep and anaesthesia (Ferrarelli et al., 2010; Massimini et al., 2005, 2012; Sarasso et al., 2015; Tononi and Massimini, 2008). Arguably, our findings reflect the earliest stages of TEP modulation as a function of alertness, with slow wave (0.5–2 Hz) dominated brain states placed at the other side of an alertness continuum. Sleep and anaesthesia-related increases in early TEP amplitude likely reflect facilitation of a stereotypical, local mode of processing (Ferrarelli et al., 2010; Massimini et al., 2005, 2012; Sarasso et al., 2015; Tononi and Massimini, 2008). We show that such a shift toward local processing starts developing while participants are still conscious of the experimental setup and able to respond behaviourally, i.e. well before they reach unresponsive sleep.

In the present study, we showed that MEP and TEP amplitudes are positively associated at a single trial level. While such a relationship has been reported previously (Fecchio et al., 2017; Petrichella et al. 2017), here we extended earlier studies by showing that the MEP and TEP association is non-linearly dependent on alertness: specifically, a reliable association was observed at Alertness Level 3 but not at Levels 1–2 or 4–5. We also observed a gradual increase in inter-trial variability of TEP amplitude across decreasing levels of alertness, whereas variability in MEP amplitude did not show a consistent change as a function of alertness. These observations suggest that the relationship between alertness levels and TMS-evoked neural responses is non-monotonic and can depend on a particular neurophysiological index as well as the level of alertness. If possible, alertness should be controlled at a single-trial level when experiments involve hundreds of trials delivered over a prolonged testing session, especially when participants keep their eyes closed. Even though standard statistical measures of central tendency can reduce the effect of alertness when carrying out within-participant contrasts, more trials are required to offset alertness-related variability, and having a reliable measure of participants’ level of alertness could reduce the length of such experiments. Furthermore, measures of central tendency cannot eliminate alertness confounds from between-participant or longitudinal comparisons. For instance, patients or older individuals might have consistently lower or higher levels of alertness compared with healthy controls.

While the change in TEP amplitude across different levels of alertness can be interpreted in terms of cortical reactivity/excitability, an alternative explanation is that TEP increases were triggered by a phase-reset of pre-TMS oscillations (Herring et al., 2015; Kawasaki et al., 2014; Thut et al., 2011). It is conceivable that a phase-reset of relatively slow theta oscillations in drowsy trials, as opposed to the faster alpha oscillations in awake trials, could underlie the increase in TEP amplitude we observed. On the other hand, even though the phase of spontaneous mu-oscillations predicts TEP amplitude, single-pulse TMS does not seem to reset the phase of mu-oscillations (Desideri et al., 2019). Clearly, more research is needed to investigate the potential role of phase-resetting in the generation of TEPs (Pellicciari et al., 2017), and in this context we argue that the transition from wake to sleep provides an ecologically valid model to study it.

Several strategies exist for dealing with changing levels of alertness during behavioural testing. If alertness decreases throughout a session, an additional factor of trial or block number could be added to a statistical model as an alertness regressor or covariate. However, we found that an initial decrease in alertness did not persist throughout the testing session, and participants tended to “oscillate” between awake and drowsy periods (see Fig. A.2), which precludes any straightforward inference of decreasing alertness over the course of a single testing session. Alternatively, reaction times (RTs) could be used as a behavioural index of alertness in active TMS experiments, as RTs typically lengthen with decreases in vigilance (Schmidt et al., 2009), increases in drowsiness (Ogilvie and Wilkinson, 1984), and following sleep deprivation (Ratcliff and Van Dongen, 2011). However, such a strategy would not be possible in passive paradigms in which participants are not required to respond (Gordon et al., 2018; Massimini et al., 2005). Furthermore, as both trial counts and reaction times are relative measures, participants who maintain high alertness throughout a session could be falsely labelled as drowsy in a proportion of trials. Arguably, concurrent EEG recording should be the gold standard for assessment of single-trial alertness, as it provides quantifiable and reliable signatures of instantaneous brain-states, including alpha- and theta-derived Hori stages of sleep onset (Hori et al., 1994). As an alternative to the tedious manual scoring of Hori stages (Alertness Levels), an automated EEG method based on wakefulness and sleep grapho-elements is available for the detection of drowsiness from EEG data (Jagannathan et al., 2018). While methods that weight the dominance of EEG theta and alpha oscillations are suitable for eyes-closed paradigms (Hori et al., 1994; Jagannathan et al., 2018), such as resting state or phosphene studies (Bonnard et al., 2016; De Graaf et al., 2017), the power of higher EEG frequencies should be considered when assessing alertness during active eyes-open experiments (Eoh et al., 2005; Kaida et al., 2006; Zhao et al., 2012). Finally, when EEG measurements are not available or feasible, TMS experiments could be carried out in short blocks of just a few minutes each (e.g., 3–5 min) and inter-block intervals could be used to assess instantaneous subjective sleepiness, for example by asking participants to undertake the 9-graded Karolinska Sleepiness Scale (Åkerstedt and Gillberg, 1990; Kaida et al., 2006). Future studies should compare other methods for capturing the contribution of fluctuating levels of alertness to data variance in TMS studies.

Here we used an eyes-closed, lights-dimmed behavioural paradigm with a long inter-pulse interval and an explicit instruction that participants could fall asleep should they feel the urge to do so, in order to promote sufficient episodes of drowsiness for statistical analysis. As such, our paradigm likely facilitated transitions from wake to sleep and induced much larger fluctuations in alertness than standard TMS-EEG-EMG setups. One implication is that researchers should exercise caution in applying our conclusions to other testing scenarios. To extend the generalisability of the current findings, future studies should employ eyes-open paradigms with more rapid stimulation to investigate the effects of alertness fluctuations on TMS-induced changes in cortical reactivity. It will also be important to investigate whether neurophysiological effects of decreasing alertness are limited to individuals who are likely to fall asleep in a situation of prolonged inactivity, as was the case for most of our participants.

While our participants used earplugs to minimise TMS-related auditory evoked potentials, and we removed auditory components and restricted TEP analyses to the time window <50 ms, traditionally known to be free from auditory contamination (Nikouline et al., 1999; Tiitinen et al., 1999), several recent TMS studies have highlighted the impact of sensory co-stimulation confounds, especially in the somatosensory domain, even at the earliest time windows (Biabani et al., 2019; Conde et al., 2019). Given the growing concern about inadvertent sensory co-stimulation in TMS experiments (Belardinelli et al., 2019; Siebner et al., 2019), the addition of a realistic sham condition would be desirable in future TEP studies, preferably with separate auditory and somatosensory conditions. As a final point, we observed that the strength of association between theta/alpha ratio and MEP slope fluctuates at a 1 Hz frequency (see Fig. A.4 A). Given the lack of previous literature on the association between theta/alpha ratio and MEP, we cannot be certain whether it is a genuine phenomenon or a statistical coincidence; hence, it should be replicated before drawing any firm conclusions.

To conclude, our findings challenge the widely held assumption that the cortex is maintained in a more or less “steady state” when participants undertake experimental investigations of perceptual, cognitive or motor function. Our findings demonstrate that spontaneously occurring fluctuations in alertness differentially modulate cortical reactivity over relatively short durations, even when participants are tested during day time hours when they would normally be awake and performing typical activities of daily living. Our study highlights the importance of controlling for spontaneous fluctuations in alertness at a single trial level in non-invasive brain stimulation studies.

## CRediT author statement

**Valdas Noreika:** Conceptualization, Methodology, Software, Formal analysis, Investigation, Data Curation, Writing - Original Draft, Writing - Review and Editing, Visualization. **Marc R. Kamke:** Conceptualization, Methodology, Investigation, Data Curation, Writing - Original Draft, Writing - Review and Editing. **Andrés Canales-Johnson:** Methodology, Software, Formal analysis, Writing - Review and Editing. **Srivas Chennu:** Methodology, Software, Formal analysis, Writing - Review and Editing. **Tristan A. Bekinschtein:** Conceptualization, Methodology, Writing - Review and Editing, Supervision, Project administration, Funding acquisition. **Jason B. Mattingley:** Conceptualization, Resources, Writing - Original Draft, Writing - Review and Editing, Supervision, Project administration, Funding acquisition.

## Declaration of Competing Interest

The authors declare no competing interests.

## Appendix Supplementary materials

Appendix A. Supplementary Figures A.1-A.11 and Tables A.1-A.3 Image, application 1

## References

[bib0001] Abrahamyan A., Clifford C.W., Ruzzoli M., Phillips D., Arabzadeh E., Harris J.A. (2011). Accurate and rapid estimation of phosphene thresholds (REPT). PLoS One.

[bib0002] Åkerstedt T., Gillberg M. (1990). Subjective and objective sleepiness in the active individual. Int. J. Neurosci..

[bib0003] Avesani M., Formaggio E., Fuggetta G., Fiaschi A., Manganotti P. (2008). Corticospinal excitability in human subjects during nonrapid eye movement sleep: single and paired-pulse transcranial magnetic stimulation study. Exp. Brain Res..

[bib0004] Bareham C.A., Manly T., Pustovaya O.V, Scott S.K., Bekinschtein T.A. (2014). Losing the left side of the world: Rightward shift in human spatial attention with sleep onset. Sci. Rep..

[bib0005] Barker A.T., Jalinous R., Freeston I.L. (1985). Non-invasive magnetic stimulation of human motor cortex. The Lancet.

[bib0006] Belardinelli P., Biabani M., Blumberger D.M., Bortoletto M., Casarotto S., David O., Ilmoniemi R.J. (2019). Reproducibility in TMS–EEG studies: a call for data sharing, standard procedures and effective experimental control. Brain Stimul..

[bib0007] Bergmann T.O., Mölle M., Schmidt M.A., Lindner C., Marshall L., Born J., Siebner H.R. (2012). EEG-guided transcranial magnetic stimulation reveals rapid shifts in motor cortical excitability during the human sleep slow oscillation. J. Neurosci..

[bib0008] Bergmann T.O., Lieb A., Zrenner C., Ziemann U. (2019). Pulsed facilitation of corticospinal excitability by the sensorimotor mu-alpha rhythm. J. Neurosci..

[bib0009] Bestmann S., Krakauer J.W. (2015). The uses and interpretations of the motor-evoked potential for understanding behaviour. Exp. Brain Res..

[bib0010] Biabani M., Fornito A., Mutanen T.P., Morrow J., Rogasch N.C. (2019). Characterizing and minimizing the contribution of sensory inputs to TMS-evoked potentials. Brain Stimul..

[bib0011] Bonnard M., Chen S., Gaychet J., Carrere M., Woodman M., Giusiano B., Jirsa V. (2016). Resting state brain dynamics and its transients: a combined TMS-EEG study. Sci. Rep..

[bib0012] Bullmore E.T., Suckling J., Overmeyer S., Rabe-Hesketh S., Taylor E., Brammer M.J. (1999). Global, voxel, and cluster tests, by theory and permutation, for a difference between two groups of structural MR images of the brain. IEEE Trans. Med. Imaging.

[bib0013] Capotosto P., Babiloni C., Romani G.L., Corbetta M. (2009). Frontoparietal cortex controls spatial attention through modulation of anticipatory alpha rhythms. J. Neurosci..

[bib0014] Chennu S., Noreika V., Gueorguiev D., Blenkmann A., Kochen S., Ibáñez A., …, Bekinschtein T.A. (2013). Expectation and attention in hierarchical auditory prediction. J. Neurosci..

[bib0015] Chung S.W., Rogasch N.C., Hoy K.E., Fitzgerald P.B. (2015). Measuring brain stimulation induced changes in cortical properties using TMS-EEG. Brain Stimul..

[bib0016] Civardi C., Boccagni C., Vicentini R., Bolamperti L., Tarletti R., Varrasi C., Cantello R. (2001). Cortical excitability and sleep deprivation: a transcranial magnetic stimulation study. J. Neurol. Neurosurg. Psychiatry.

[bib0017] Cohen J. (1988). Statistical Power Analysis for the Behavioral Sciences.

[bib0018] Cohen M.X. (2014). Analyzing Neural Time Series Data: Theory and Practice.

[bib0019] Conde V., Tomasevic L., Akopian I., Stanek K., Saturnino G.B., Thielscher A., Siebner H.R. (2019). The non-transcranial TMS-evoked potential is an inherent source of ambiguity in TMS-EEG studies. Neuroimage.

[bib0020] De Gee J.W., Colizoli O., Kloosterman N.A., Knapen T., Nieuwenhuis S., Donner T.H. (2017). Dynamic modulation of decision biases by brainstem arousal systems. eLife.

[bib0021] De Gennaro L., Marzano C., Veniero D., Moroni F., Fratello F., Curcio G., Rossini P.M. (2007). Neurophysiological correlates of sleepiness: A combined TMS and EEG study. Neuroimage.

[bib0022] De Graaf T.A., Duecker F., Stankevich Y., Ten Oever S., Sack A.T. (2017). Seeing in the dark: Phosphene thresholds with eyes open versus closed in the absence of visual inputs. Brain Stimul..

[bib0023] Delorme A., Makeig S. (2004). EEGLAB: An open source toolbox for analysis of single-trial EEG dynamics including independent component analysis. J. Neurosci. Methods.

[bib0024] Desideri D., Zrenner C., Ziemann U., Belardinelli P. (2019). Phase of sensorimotor μ‐oscillation modulates cortical responses to transcranial magnetic stimulation of the human motor cortex. J. Physiol..

[bib0025] Dugué L., VanRullen R. (2017). Transcranial magnetic stimulation reveals intrinsic perceptual and attentional rhythms. Front. Neurosci..

[bib0026] Ellaway P.H., Davey N.J., Maskill D.W., Rawlinson S.R., Lewis H.S., Anissimova N.P. (1998). Variability in the amplitude of skeletal muscle responses to magnetic stimulation of the motor cortex in man. Electroencephalogr. Clin. Neurophys..

[bib0027] Eoh H.J., Chung M.K., Kim S.H. (2005). Electroencephalographic study of drowsiness in simulated driving with sleep deprivation. Int. J. Ind. Ergonom..

[bib0028] Fecchio M., Pigorini A., Comanducci A., Sarasso S., Casarotto S., Premoli I., Ferrarelli F. (2017). The spectral features of EEG responses to transcranial magnetic stimulation of the primary motor cortex depend on the amplitude of the motor evoked potentials. PloS One.

[bib0029] Ferrarelli F., Massimini M., Sarasso S., Casali A., Riedner B.A., Angelini G., Pearce R.A. (2010). Breakdown in cortical effective connectivity during midazolam-induced loss of consciousness. Proc. Natl. Acad. Sci..

[bib0030] Goldsworthy M.R., Hordacre B., Ridding M.C. (2016). Minimum number of trials required for within-and between-session reliability of TMS measures of corticospinal excitability. Neuroscience.

[bib0031] Gordon P.C., Zrenner C., Desideri D., Belardinelli P., Zrenner B., Brunoni A.R., Ziemann U. (2018). Modulation of cortical responses by transcranial direct current stimulation of dorsolateral prefrontal cortex: a resting-state EEG and TMS-EEG study. Brain Stimul..

[bib0032] Grosse P., Khatami R., Salih F., Kuhn A., Meyer B.U. (2002). Corticospinal excitability in human sleep as assessed by transcranial magnetic stimulation. Neurology.

[bib0033] Herring J.D., Thut G., Jensen O., Bergmann T.O. (2015). Attention modulates TMS-locked alpha oscillations in the visual cortex. J. Neurosci..

[bib0034] Hershner S.D., Chervin R.D. (2014). Causes and consequences of sleepiness among college students. Nat. Sci. Sleep.

[bib0035] Hess C.W., Mills K.R., Murray N.M.F., Schriefer T.N. (1987). Excitability of the human motor cortex is enhanced during REM sleep. Neurosci. Lett..

[bib0036] Holm S. (1979). A simple sequentially rejective multiple test procedure. Scand. J. Stat..

[bib0037] Hori T., Hayashi M., Morikawa T., Ogilvie R.D., Harsh J.R. (1994). Topographical EEG changes and the hypnagogic experience. Sleep Onset: Normal and Abnormal Processes.

[bib0038] Huber R., Mäki H., Rosanova M., Casarotto S., Canali P., Casali A.G., Massimini M. (2013). Human cortical excitability increases with time awake. Cereb. Cortex.

[bib0039] Hussain S.J., Claudino L., Bönstrup M., Norato G., Cruciani G., Thompson R., Cohen L.G. (2018). Sensorimotor oscillatory phase–power interaction gates resting human corticospinal output. Cereb. Cortex.

[bib0040] Ikoma K., Samii A., Mercuri B., Wassermann E.M., Hallett M. (1996). Abnormal cortical motor excitability in dystonia. Neurology.

[bib0041] Ilmoniemi R.J., Virtanen J., Ruohonen J., Karhu J., Aronen H.J., Näätänen R., Katila T. (1997). Neuronal responses to magnetic stimulation reveal cortical reactivity and connectivity. Neuroreport.

[bib0042] Iscan Z., Nazarova M., Fedele T., Blagovechtchenski E., Nikulin V.V. (2016). Pre-stimulus alpha oscillations and inter-subject variability of motor evoked potentials in single-and paired-pulse TMS paradigms. Front. Hum. Neurosci..

[bib0043] Jagannathan S.R., Ezquerro-Nassar A., Jachs B., Pustovaya O.V., Bareham C.A., Bekinschtein T.A. (2018). Tracking wakefulness as it fades: micro-measures of alertness. NeuroImage.

[bib0044] Johns M.W. (1991). A new method for measuring daytime sleepiness: the Epworth sleepiness scale. Sleep.

[bib0045] Kaida K., Takahashi M., Åkerstedt T., Nakata A., Otsuka Y., Haratani T., Fukasawa K. (2006). Validation of the Karolinska sleepiness scale against performance and EEG variables. Clin. Neurophysiol..

[bib0046] Kaur G., Singh A. (2017). Excessive daytime sleepiness and its pattern among Indian college students. Sleep Med..

[bib0047] Kawasaki M., Uno Y., Mori J., Kobata K., Kitajo K. (2014). Transcranial magnetic stimulation-induced global propagation of transient phase resetting associated with directional information flow. Front. Hum. Neurosci..

[bib0048] Keil J., Timm J., SanMiguel I., Schulz H., Obleser J., Schönwiesner M. (2014). Cortical brain states and corticospinal synchronization influence TMS-evoked motor potentials. J. Neurophysiol..

[bib0049] Kiers L., Cros D., Chiappa K.H., Fang J. (1993). Variability of motor potentials evoked by transcranial magnetic stimulation. Electroencephalogr. Clin. Neurophysiol..

[bib0050] Kleiner, M., Brainard, D., Pelli, D., 2007. What's new in Psychtoolbox-3? Perception 36 ECVP Abstract Supplement.

[bib0051] Ly J.Q., Gaggioni G., Chellappa S.L., Papachilleos S., Brzozowski A., Borsu C., Archer S.N. (2016). Circadian regulation of human cortical excitability. Nat. Commun..

[bib0053] Maeda F., Gangitano M., Thall M., Pascual-Leone A. (2002). Inter-and intra-individual variability of paired-pulse curves with transcranial magnetic stimulation (TMS). Clin. Neurophysiol..

[bib0054] Madsen K.H., Karabanov A.N., Krohne L.G., Safeldt M.G., Tomasevic L., Siebner H.R. (2019). No trace of phase: corticomotor excitability is not tuned by phase of pericentral mu-rhythm. Brain Stimul..

[bib0055] Mäki H., Ilmoniemi R.J. (2010). EEG oscillations and magnetically evoked motor potentials reflect motor system excitability in overlapping neuronal populations. Clin. Neurophysiol..

[bib0056] Manganotti P., Palermo A., Patuzzo S., Zanette G., Fiaschi A. (2001). Decrease in motor cortical excitability in human subjects after sleep deprivation. Neurosci. Lett..

[bib0057] Manganotti P., Fuggetta G., Fiaschi A. (2004). Changes of motor cortical excitability in human subjects from wakefulness to early stages of sleep: a combined transcranial magnetic stimulation and electroencephalographic study. Neurosci. Lett..

[bib0058] Manganotti P., Bongiovanni L.G., Fuggetta G., Zanette G., Fiaschi A. (2006). Effects of sleep deprivation on cortical excitability in patients affected by juvenile myoclonic epilepsy: a combined transcranial magnetic stimulation and EEG study. J. Neurol. Neurosurg. Psychiatry.

[bib0059] Maris E., Oostenveld R. (2007). Nonparametric statistical testing of EEG- and MEG-data. J. Neurosci. Methods.

[bib0060] Massimini M., Ferrarelli F., Huber R., Esser S.K., Singh H., Tononi G. (2005). Breakdown of cortical effective connectivity during sleep. Science.

[bib0061] Massimini M., Ferrarelli F., Esser S.K., Riedner B.A., Huber R., Murphy M., Tononi G. (2007). Triggering sleep slow waves by transcranial magnetic stimulation. Proc. Natl. Acad. Sci..

[bib0062] Massimini M., Ferrarelli F., Sarasso S., Tononi G. (2012). Cortical mechanisms of loss of consciousness: insight from TMS/EEG studies. Arch. Italiennes Biol..

[bib0063] Nikouline V., Ruohonen J., Ilmoniemi R.J. (1999). The role of the coil click in TMS assessed with simultaneous EEG. Clin. Neurophys..

[bib0064] Nittono H., Momose D., Hori T. (1999). Gradual changes of mismatch negativity during the sleep onset period. Sleep Res. Online.

[bib0065] Noreika V., Canales-Johnson A., Harrison W.J., Johnson A., Arnatkevičiūtė A., Koh J., Bekinschtein T.A. (2020). Wakefulness fluctuations elicit behavioural and neural reconfiguration of awareness. bioRxiv.

[bib0066] Ogata K., Nakazono H., Uehara T., Tobimatsu S. (2019). Prestimulus cortical EEG oscillations can predict the excitability of the primary motor cortex. Brain Stimul..

[bib0067] Ogilvie R.D. (2001). The process of falling asleep. Sleep Med. Rev..

[bib0068] Ogilvie R.D., Wilkinson R.T. (1984). The detection of sleep onset: behavioral and physiological convergence. Psychophysiology.

[bib0069] Oldfield R.C. (1971). The assessment and analysis of handedness: the Edinburgh inventory. Neuropsychologia.

[bib0070] Oostenveld R., Fries P., Maris E., Schoffelen J.M. (2011). FieldTrip: open source software for advanced analysis of MEG, EEG, and invasive electrophysiological data. Comput. Intell. Neurosci..

[bib0071] Pellicciari M.C., Veniero D., Miniussi C. (2017). Characterizing the cortical oscillatory response to TMS pulse. Front. Cellular Neurosci..

[bib0072] Petrichella S., Johnson N., He B. (2017). The influence of corticospinal activity on TMS-evoked activity and connectivity in healthy subjects: a TMS-EEG study. PLoS One.

[bib0073] Pratt H., Starr A., Michalewski H.J., Bleich N., Mittelman N. (2008). The auditory P50 component to onset and offset of sound. Clin. Neurophysiol..

[bib0074] Premoli I., Castellanos N., Rivolta D., Belardinelli P., Bajo R., Zipser C., Ziemann U. (2014). TMS-EEG signatures of GABAergic neurotransmission in the human cortex. J. Neurosci..

[bib0075] Ratcliff R., Van Dongen H.P. (2011). Diffusion model for one-choice reaction-time tasks and the cognitive effects of sleep deprivation. Proc. Natl. Acad. Scie..

[bib0076] Rodrigues R.N.D., Viegas C.A.A., Abreu e Silva A.A.A., Tavares P. (2002). Daytime sleepiness and academic performance in medical students. Arq. Neuro-psiquiatr..

[bib0077] Rogasch N.C., Thomson R.H., Farzan F., Fitzgibbon B.M., Bailey N.W., Hernandez-Pavon J.C., Fitzgerald P.B. (2014). Removing artefacts from TMS-EEG recordings using independent component analysis: importance for assessing prefrontal and motor cortex network properties. Neuroimage.

[bib0078] Rogasch N.C., Sullivan C., Thomson R.H., Rose N.S., Bailey N.W., Fitzgerald P.B., Hernandez-Pavon J.C. (2017). Analysing concurrent transcranial magnetic stimulation and electroencephalographic data: a review and introduction to the open-source TESA software. Neuroimage.

[bib0079] Romei V., Brodbeck V., Michel C., Amedi A., Pascual-Leone A., Thut G. (2008). Spontaneous fluctuations in posterior α-band EEG activity reflect variability in excitability of human visual areas. Cereb. Cortex.

[bib0080] Rösler K.M., Roth D.M., Magistris M.R. (2008). Trial-to-trial size variability of motor-evoked potentials. A study using the triple stimulation technique. Exp. Brain Res..

[bib0081] Rossi S., Hallett M., Rossini P.M., Pascual-Leone A., Avanzini G., Bestmann S., Ziemann U. (2009). Safety, ethical considerations, and application guidelines for the use of transcranial magnetic stimulation in clinical practice and research. Clin. Neurophysiol..

[bib0082] Rossini P.M., Barker A.T., Berardelli A., Caramia M.D., Caruso G., Cracco R.Q., Tomberg C. (1994). Non-invasive electrical and magnetic stimulation of the brain, spinal cord and roots: basic principles and procedures for routine clinical application. Report of an IFCN committee. Electroencephalogr. Clin. Neurophysiol..

[bib0083] Sale M.V., Ridding M.C., Nordstrom M.A. (2007). Factors influencing the magnitude and reproducibility of corticomotor excitability changes induced by paired associative stimulation. Exp. Brain Res..

[bib0084] Samii A., Wassermann E.M., Ikoma K., Mercuri B., Hallett M. (1996). Characterization of postexercise facilitation and depression of motor evoked potentials to transcranial magnetic stimulation. Neurology.

[bib0085] Sarasso S., Boly M., Napolitani M., Gosseries O., Charland-Verville V., Casarotto S., Rex S. (2015). Consciousness and complexity during unresponsiveness induced by propofol, xenon, and ketamine. Curr. Biol..

[bib0086] Sauseng P., Klimesch W., Gerloff C., Hummel F.C. (2009). Spontaneous locally restricted EEG alpha activity determines cortical excitability in the motor cortex. Neuropsychologia.

[bib0087] Schmidt E.A., Schrauf M., Simon M., Fritzsche M., Buchner A., Kincses W.E. (2009). Drivers’ misjudgement of vigilance state during prolonged monotonous daytime driving. Accid. Anal. Prev..

[bib0088] Schulz H., Übelacker T., Keil J., Müller N., Weisz N. (2013). Now I am ready—now I am not: the influence of pre-TMS oscillations and corticomuscular coherence on motor-evoked potentials. Cereb. Cortex.

[bib0089] Schutter D.J., Hofman D., Hoppenbrouwers S.S., Kenemans J.L. (2011). Corticospinal state variability and hemispheric asymmetries in motivational tendencies. Biol. Psychol..

[bib0090] Siebner H.R., Conde V., Tomasevic L., Thielscher A., Bergmann T.O. (2019). Distilling the essence of TMS-evoked EEG potentials (TEPs): A call for securing mechanistic specificity and experimental rigor. Brain Stimul..

[bib0091] Sommer M., Wu T., Tergau F., Paulus W. (2002). Intra-and interindividual variability of motor responses to repetitive transcranial magnetic stimulation. Clin. Neurophysiol..

[bib0092] Tagliazucchi E., Laufs H. (2014). Decoding wakefulness levels from typical fMRI resting-state data reveals reliable drifts between wakefulness and sleep. Neuron.

[bib0093] Tanaka H., Hayashi M., Hori T. (1997). Topographical characteristics and principal component structure of the hypnagogic EEG. Sleep.

[bib0094] Thies M., Zrenner C., Ziemann U., Bergmann T.O. (2018). Sensorimotor mu-alpha power is positively related to corticospinal excitability. Brain Stimul..

[bib0095] Thut G., Veniero D., Romei V., Miniussi C., Schyns P., Gross J. (2011). Rhythmic TMS causes local entrainment of natural oscillatory signatures. Curr. Biol..

[bib0096] Tiitinen H., Virtanen J., Ilmoniemi R.J., Kamppuri J., Ollikainen M., Ruohonen J., Näätänen R. (1999). Separation of contamination caused by coil clicks from responses elicited by transcranial magnetic stimulation. Clin. Neurophysiol..

[bib0097] Tononi G., Massimini M. (2008). Why does consciousness fade in early sleep. Ann. N.Y. Acad. Sci..

[bib0098] Valero-Cabré A., Amengual J.L., Stengel C., Pascual-Leone A., Coubard O.A. (2017). Transcranial magnetic stimulation in basic and clinical neuroscience: a comprehensive review of fundamental principles and novel insights. Neurosci. Biobehav. Rev..

[bib0099] van Dijk H., Schoffelen J.-M., Oostenveld R., Jensen O. (2008). Prestimulus oscillatory activity in the alpha band predicts visual discrimination ability. J. Neurosci..

[bib0100] Yang C.M., Wu C.H., Hsieh M.H., Liu M.H., Lu F.H. (2003). Coping with sleep disturbances among young adults: a survey of first-year college students in Taiwan. Behav. Med..

[bib0101] Zailinawati A.H., Teng C.L., Chung Y.C., Teow T.L., Lee P.N., Jagmohni K.S. (2009). Daytime sleepiness and sleep quality among Malaysian medical students. Med. J. Malaysia.

[bib0102] Zarkowski P., Shin C.J., Dang T., Russo J., Avery D. (2006). EEG and the variance of motor evoked potential amplitude. Clin. EEG Neurosci..

[bib0103] Ziemann U. (2017). Thirty years of transcranial magnetic stimulation: where do we stand. Exp. Brain Res..

[bib0104] Zrenner C., Desideri D., Belardinelli P., Ziemann U. (2018). Real-time EEG-defined excitability states determine efficacy of TMS-induced plasticity in human motor cortex. Brain Stimul..

[bib0105] Zhao C., Zhao M., Liu J., Zheng C. (2012). Electroencephalogram and electrocardiograph assessment of mental fatigue in a driving simulator. Accid. Anal. Prev..

