# Supplementary material for: Alertness fluctuations when performing a task modulate cortical evoked responses to transcranial magnetic stimulation
Source: Neuroimage. 2020 Dec;223:117305. doi: 10.1016/j.neuroimage.2020.117305 (PMC7762840; doi:10.1016/j.neuroimage.2020.117305)
Supplement: Supplementary file 1 — Appendix A. Supplementary Figures A.1-A.11 and Tables A.1-A.3 [file mmc1.docx]

**Title:** Alertness fluctuations when performing a task during wake-to-sleep transition modulate cortical evoked responses to transcranial magnetic stimulation

**Authors:** Valdas Noreika, Marc R. Kamke, Andrés Canales-Johnson, Srivas Chennu, Tristan A. Bekinschtein, Jason B. Mattingley

**Appendix A - Supplementary Figures A.1-A.11 and Tables A.1-A.3**


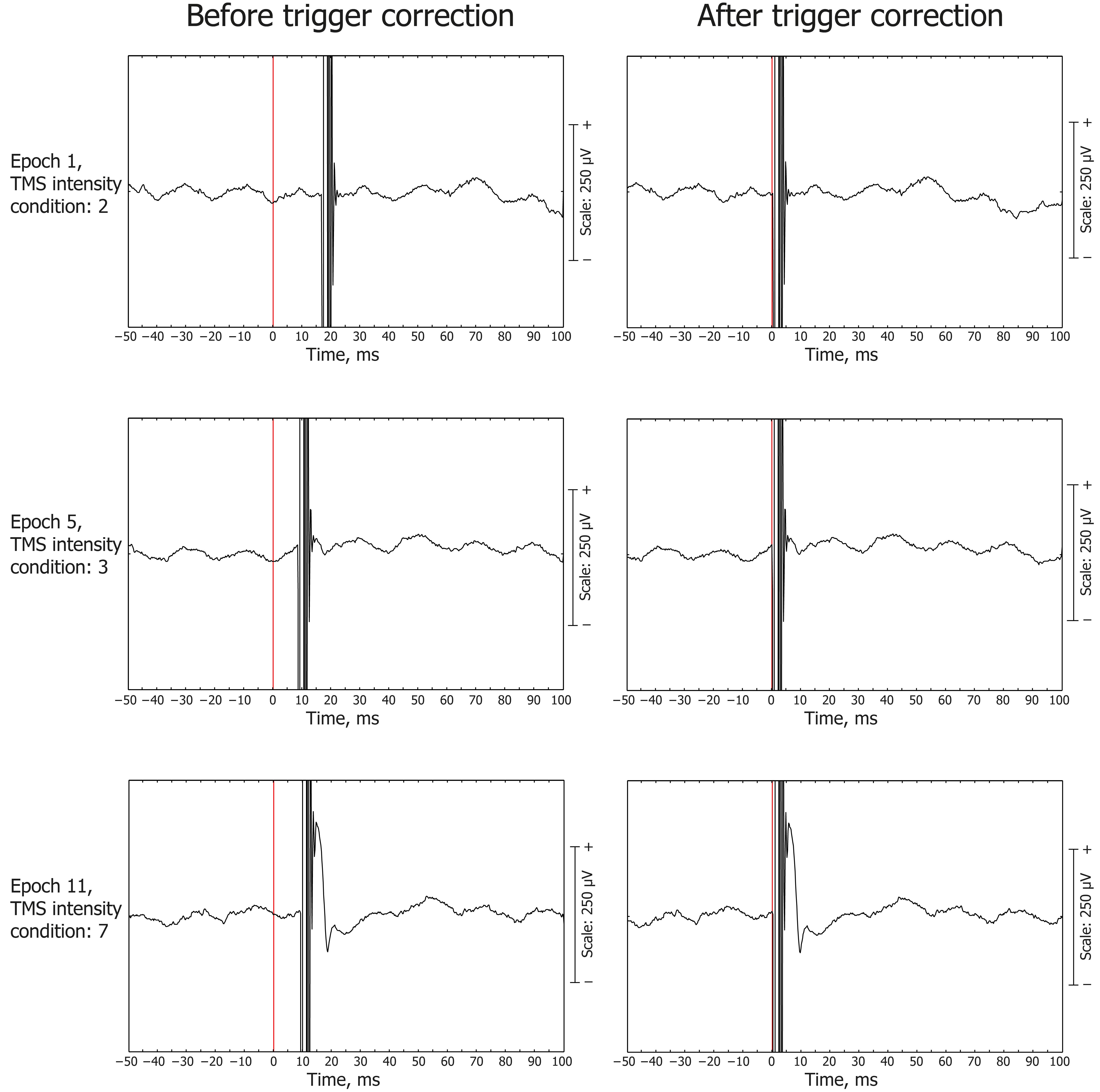


**Figure A.1 | Temporal mismatch between EEG markers of TMS pulses and actual discharge of the TMS coil.** Three example EEG epochs segmented around the TMS marker, depicted as a red vertical line, in a -50 ms to 100 ms time window (data from Participant 1). Plots on the left side indicate a time-varying delay between the marker and the TMS artefact, whereas plots on the right side demonstrate the same epochs after automatic marker realignment. Please note that a relatively long delay could occur even at low TMS intensities, e.g., Epoch 1 in the top row.


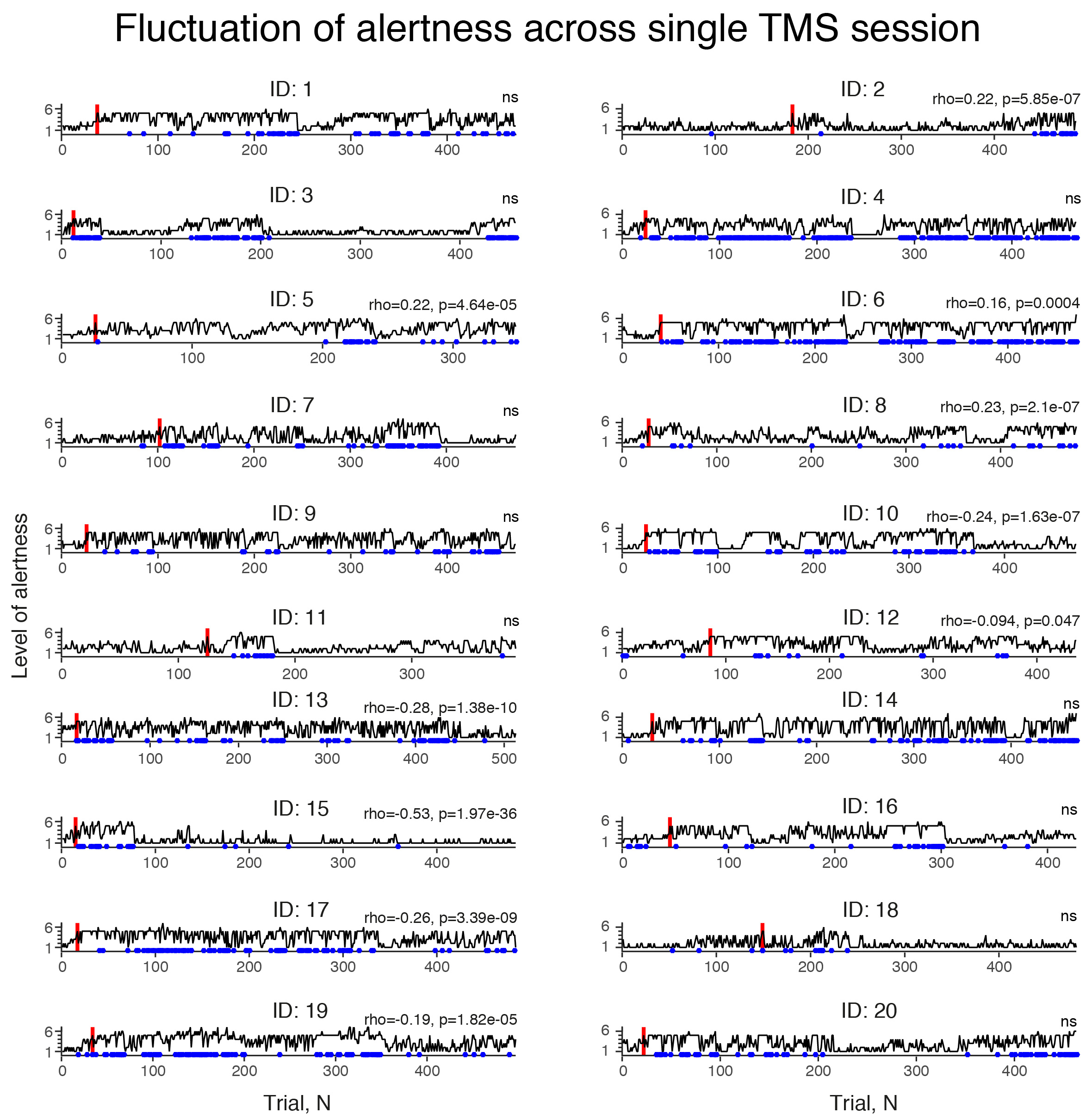


**Figure A.2 | Individual differences in levels of alertness across a single TMS session.** Visualisation of the alertness level (vertical axis) shown for the entire TMS testing session (horizontal axis). Each subplot represents a different participant (indicated by ID number). Red vertical lines depict the first trial within a session scored as Alertness Level 5. Blue dots indicate unresponsive trials. Results of Spearman rank order correlation tests between Alertness Level and trial number are presented in the top right corner of each subplot (ns=not significant).


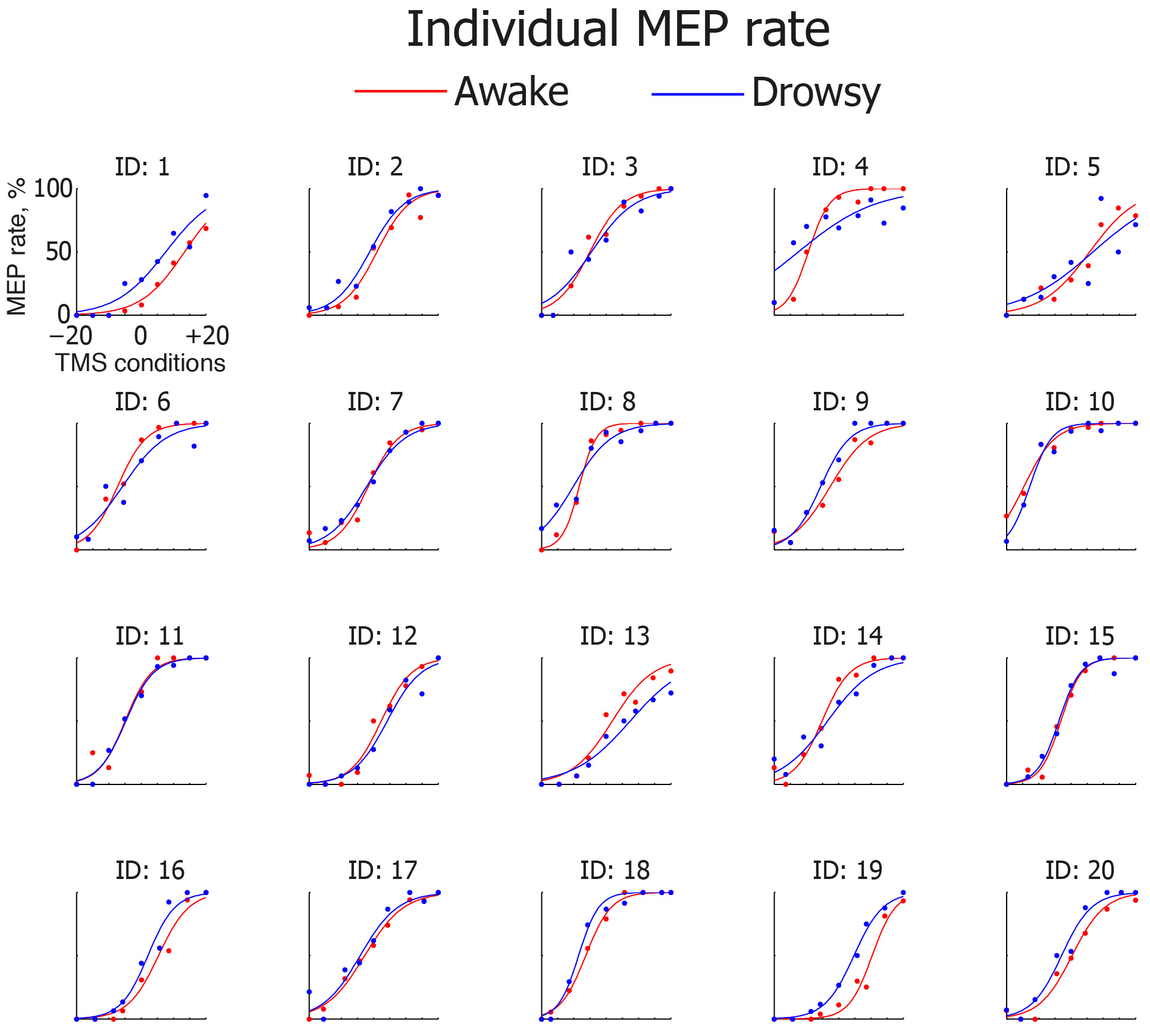


**Figure A.3 | Individual rate of motor evoked potentials (MEPs) as a function of transcranial magnetic stimulation (TMS) intensity in θ/α-defined awake and drowsy states.** Percentage of trials with MEPs above threshold value of 50μV, calculated separately for the awake and drowsy trials across 9 TMS conditions centred on individual motor threshold (0%). Sigmoidal functions are fitted to the awake (red) and drowsy (blue) conditions separately for each individual (N=20).


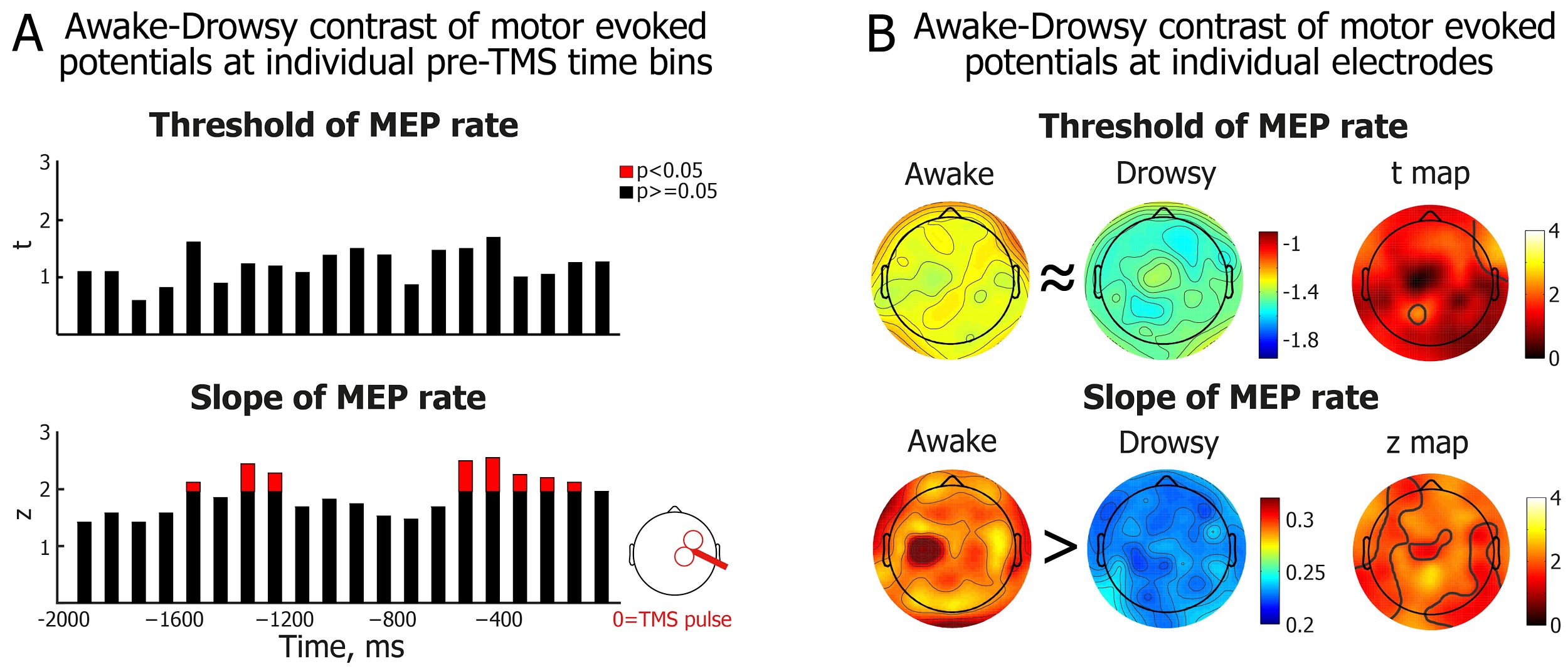


**Figure A.4 | Temporal and spatial spread of the alertness-dependent modulation of motor evoked potentials.** (A) Difference of the MEP sigmoid thresholds (upper panel) and slopes (lower panel) between θ/α-defined awake and drowsy conditions. Alertness states were measured and contrasted separately for each of the 20 time bins in steps of 100 ms across a -2000 to 0 ms pre-TMS time window. Electroencephalography (EEG) spectral power was averaged over all electrodes. (B) Difference in MEP sigmoid thresholds (upper row) and slopes (lower row) between θ/α-defined awake and drowsy conditions. Alertness states were measured and contrasted separately for each of the 63 EEG electrodes. EEG spectral power is averaged over a -2000 to 0 ms pre-stimulation time window.


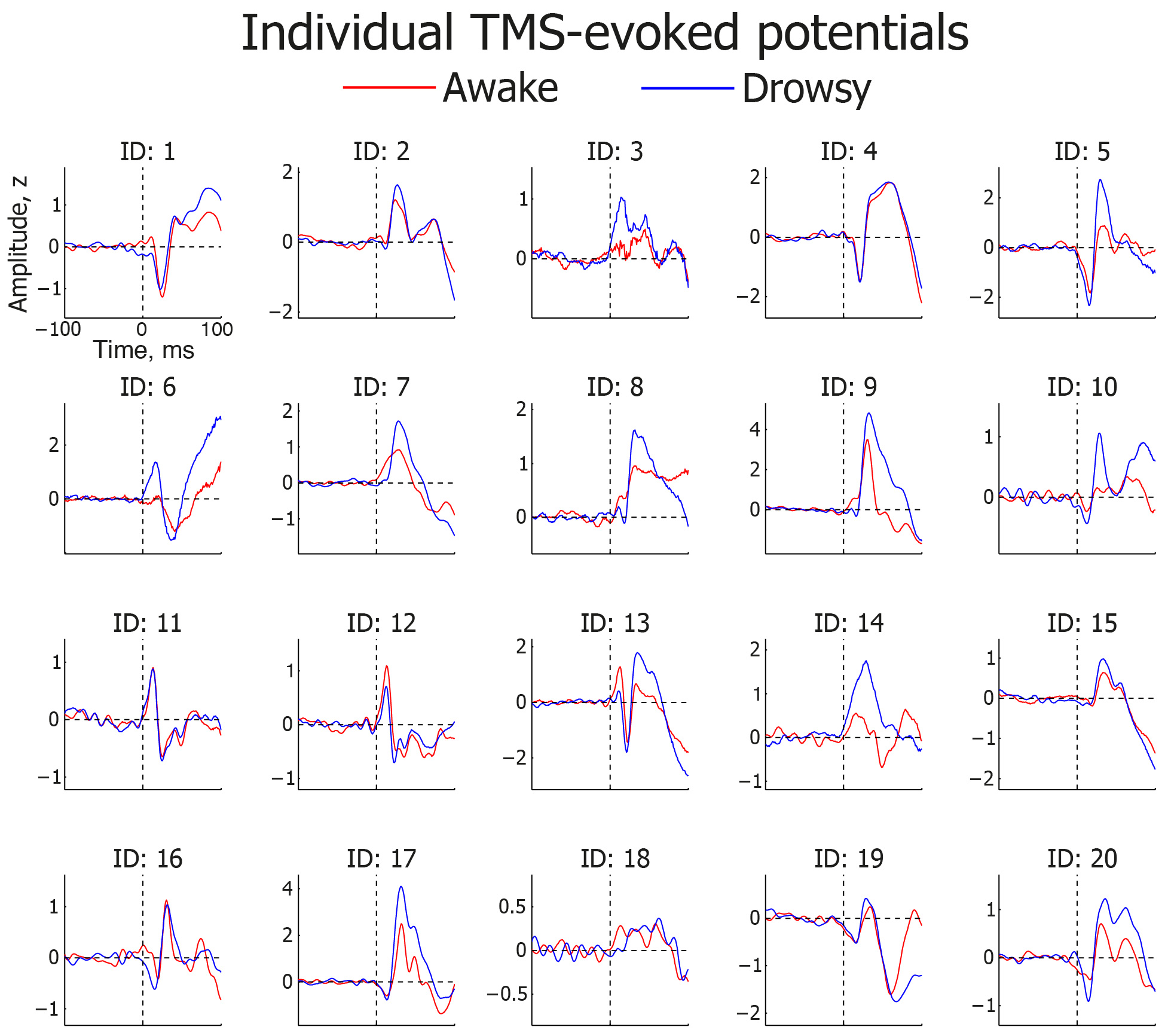


**Figure A.5 | Individual transcranial magnetic stimulation-triggered cortical reactivity potentials (TEPs) in θ/α-defined awake and drowsy states.** TEPs averaged across 4 EEG electrodes within a region-of-interest (ROI) beneath the TMS coil. Awake (red) and drowsy (blue) TEP waveforms are depicted separately for each individual (N=20).


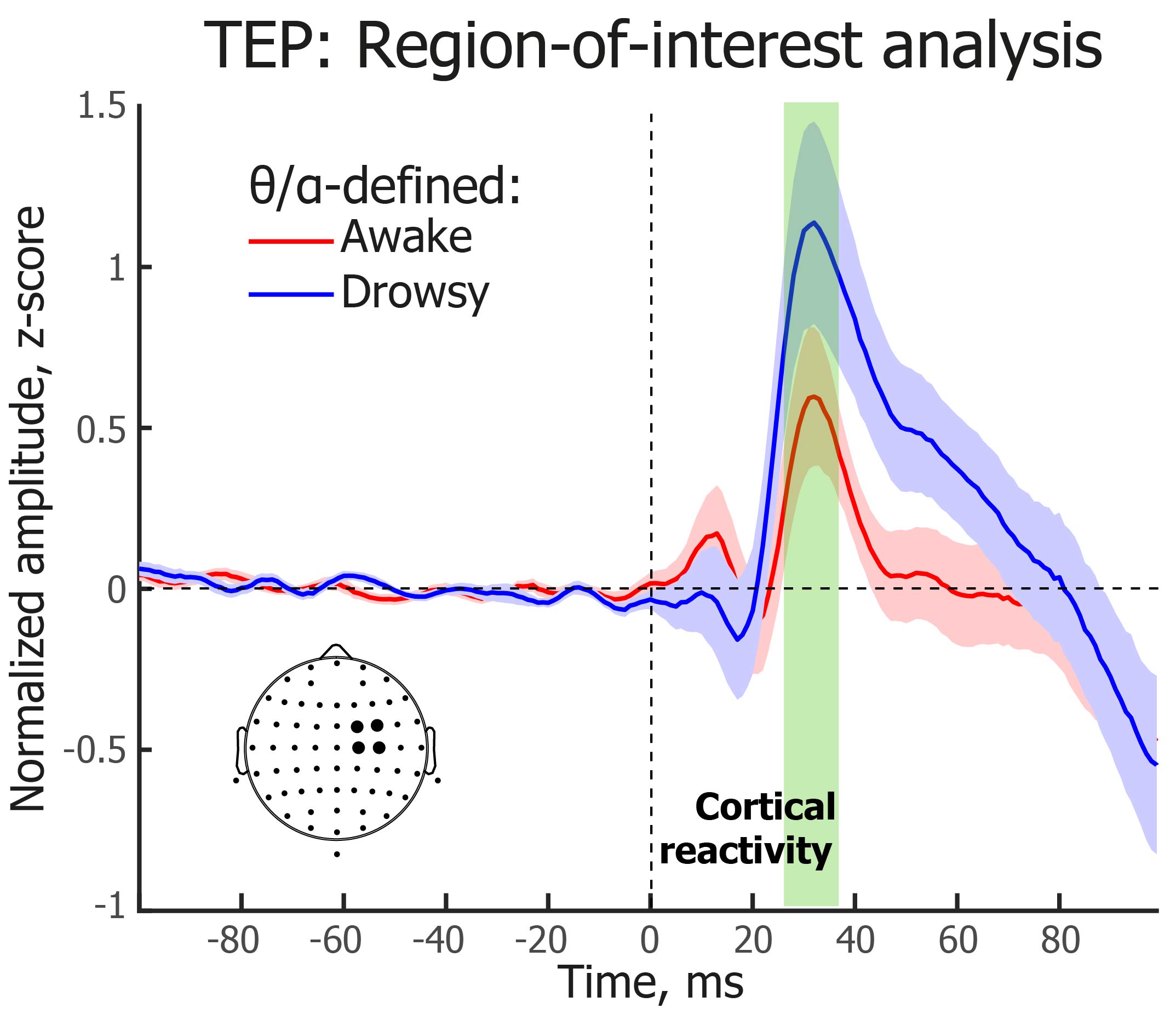


**Figure A.6 | Transcranial magnetic stimulation-triggered cortical reactivity potentials (TEPs) in awake and drowsy states: Control analysis with trial numbers matched by TMS intensity condition between states of alertness, separately for each participant.** Time course of electroencephalography (EEG) potentials averaged over 4 EEG electrodes beneath the TMS coil in the θ/α-defined awake (red) and drowsy (blue) trials. The main TEP analysis pipeline was followed here, with auditory independent components removed from EEG data. Green shaded area highlights the cortical reactivity time window (26-36 ms). Only behaviourally responsive trials are included in the analysis shown in this and other subplots. 0 ms corresponds to the time of the TMS pulse. Red and blue shading depicts standard error of the mean (SEM). There was a significant increase in TEP amplitude in the drowsy state relative to the awake state (t(19)=3.978, p=0.00081, d=0.479).


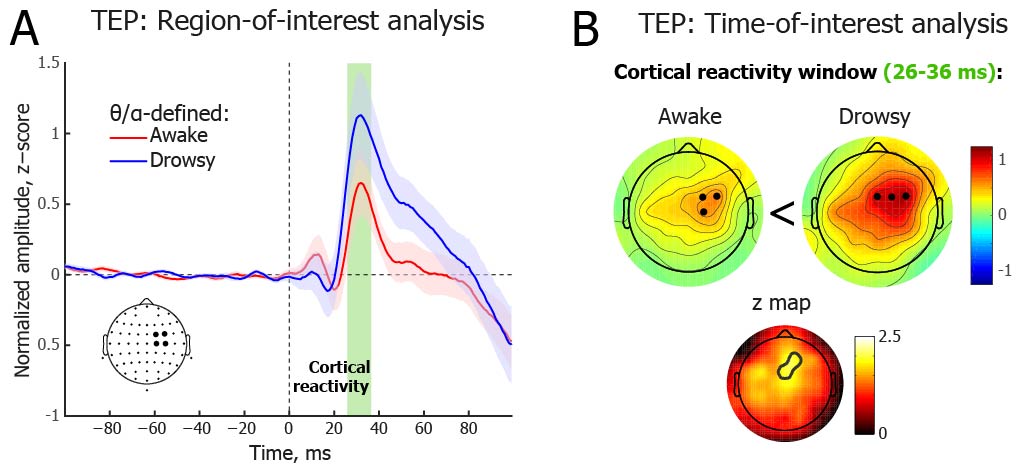


**Figure A.7 | Transcranial magnetic stimulation-triggered cortical reactivity potentials (TEPs) in the awake and drowsy states: Control analysis with MEP peak-to-peak amplitude matched between states of alertness, separately for each participant.** Time course of electroencephalography (EEG) potentials averaged over 4 EEG electrodes beneath the TMS coil in the θ/α-defined awake (red) and drowsy (blue) trials. The main TEP analysis pipeline was followed here, with auditory independent components removed from the EEG data. Green shaded area highlights the cortical reactivity time window (26-36 ms). Only behaviourally responsive trials are included in the analysis shown in this and other subplots. 0 ms corresponds to the time of the TMS pulse. Red and blue shading depicts standard error of the mean (SEM). There was a significant increase in TEP amplitude in the drowsy state relative to the awake state (t(19)=3.983, p=0.0008, d=0.432).


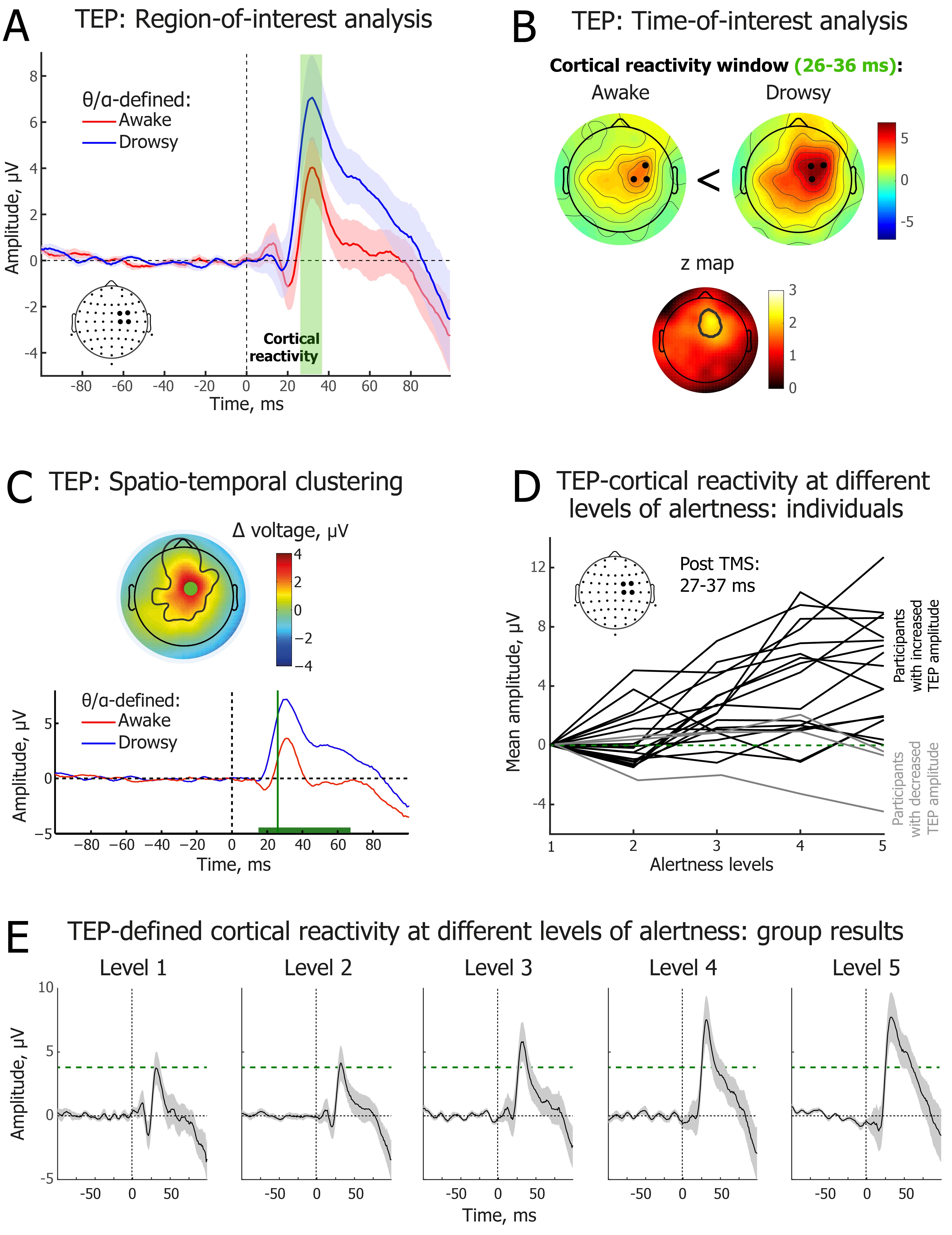


**Figure A.8 | Transcranial magnetic stimulation-triggered cortical reactivity potentials (TEPs) across different levels of alertness: Control analysis without auditory independent components in the voltage domain.** (A) Time course of electroencephalography (EEG) potentials averaged over 4 EEG electrodes beneath the TMS coil in the θ/α-defined awake (red) and drowsy (blue) trials. Green shaded area highlights the cortical reactivity time window (26-36 ms). Only behaviourally responsive trials are included in the analysis shown in this and other subplots. 0 ms corresponds to the time of the TMS pulse. Red and blue shading depicts standard error of the mean (SEM). There was a significant increase in TEP amplitude in the drowsy state relative to the awake state (t(19)=3.83, p=0.0011, d=0.47). (B) Topographical distribution of the early TEP mean peak at 26-36 ms post-TMS pulse in the θ/α-defined awake (upper left) and drowsy (upper right) states. Black dots indicate locations of three EEG electrodes with the maximal amplitude in the map. Non-parametric z map (below) reveals region significantly different between awake and drowsy states. (C) 0-100 ms data-driven spatio-temporal clustering of EEG potentials post-TMS pulse between θ/α-defined awake (red) and drowsy (blue) states. TEP amplitude was significantly higher in drowsy trials in a 15-67 ms time window (cluster peak: 26 ms, t=4594.62, p=0.002). The green horizontal line depicts the time window of significant difference. The electrode with the largest difference between awake and drowsy states is marked as a green dot in the topographic voltage map, and its waveforms are plotted below. The black contours within the map show the electrodes with statistically significant differences (cluster). The topographic voltage map is at the peak difference between awake and drowsy states. (D) Individual-level dynamics of TEP cortical reactivity peak amplitude across Alertness Levels 1-5 (TEP amplitude averaged over 27-37 ms across 4 electrodes beneath the TMS coil). Amplitude is shown relative to Alertness Level 1 (green dashed line). Black lines represent participants with higher TEP amplitude at Alertness Level 5 relative to Alertness Level 1 (N=17); grey lines represent participants with lower TEP amplitude at Alertness Level 5 relative to Alertness Level 1 (N=3). (E) Group-level dynamics of TEP waveforms across Alertness Levels 1-5 (TEPs averaged over 4 electrodes beneath the TMS coil). Horizontal green dashed line delineates TEP cortical reactivity peak at 32 ms post-TMS at Alertness Level 1. A linear trend of increasing TEP amplitude was observed across Alertness Levels 1-5 (F(1,19)=19.66, p=0.0003, partial η^2^=0.51).


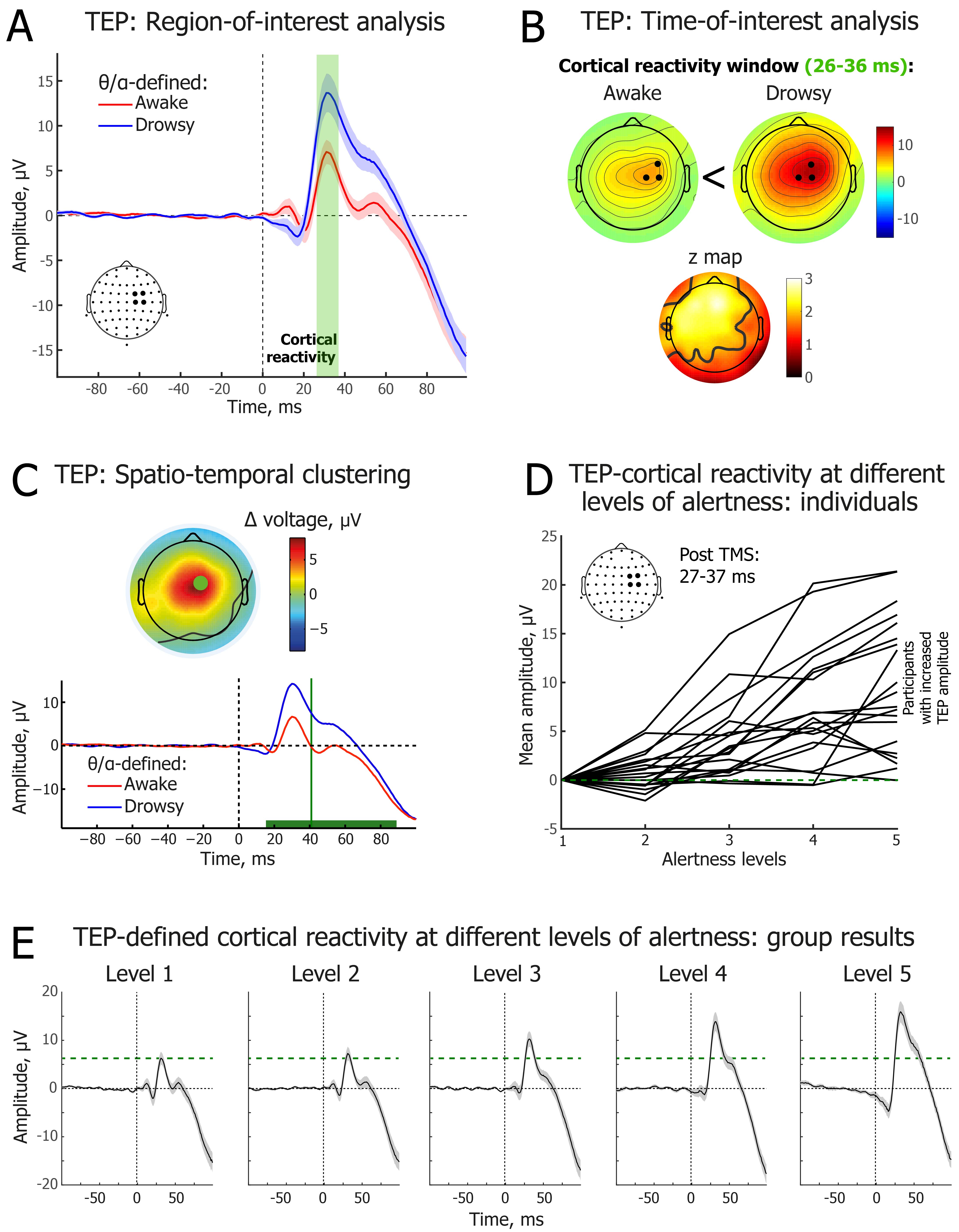


**Figure A.9 | Transcranial magnetic stimulation-triggered cortical reactivity potentials (TEPs) across different levels of alertness: Control analysis with auditory independent components in the voltage domain.** (A) Time course of electroencephalography (EEG) potentials averaged over 4 EEG electrodes beneath the TMS coil in the θ/α-defined awake (red) and drowsy (blue) trials. Green shaded area highlights the cortical reactivity time window (26-36 ms). Only behaviourally responsive trials are included in the analysis shown in this and other subplots. 0 ms corresponds to the time of the TMS pulse. Red and blue shading depicts standard error of the mean (SEM). There was a significant increase in TEP amplitude in the drowsy state relative to the awake state (t(19)=4.94, p=0.00009, d=0.86). (B) Topographical distribution of the early TEP mean peak at 26-36 ms post-TMS pulse in the θ/α-defined awake (upper left) and drowsy (upper right) states. Black dots indicate locations of three EEG electrodes with the maximal amplitude in the map. Non-parametric z map (below) reveals region significantly different between awake and drowsy states. (C) 0-100 ms data-driven spatio-temporal clustering of EEG potentials post-TMS pulse between θ/α-defined awake (red) and drowsy (blue) states. TEP amplitude was significantly higher in drowsy trials in a 15-90 ms time window (cluster peak: 41 ms, t=10696.05, p=0.001). The green horizontal line depicts the time window of significant difference. The electrode with the largest difference between awake and drowsy states is marked as a green dot in the topographic voltage map, and its waveforms are plotted below. The black contours within the map show the electrodes with statistically significant differences (cluster). The topographic voltage map is at the peak difference between awake and drowsy states. (D) Individual-level dynamics of TEP cortical reactivity peak amplitude across Alertness Levels 1-5 (TEP amplitude averaged over 27-37 ms across 4 electrodes beneath the TMS coil). Amplitude is shown relative to Alertness Level 1 (green dashed line). Black lines represent participants with higher TEP amplitude at Alertness Level 5 relative to Alertness Level 1 (N=19); grey lines represent participants with lower TEP amplitude at Alertness Level 5 relative to Alertness Level 1 (none). (E) Group-level dynamics of TEP waveforms across Alertness Levels 1-5 (TEPs averaged over 4 electrodes beneath the TMS coil). Horizontal green dashed line delineates TEP cortical reactivity peak at 32 ms post-TMS at Alertness Level 1. A linear trend of increasing TEP amplitude was observed across Alertness Levels 1-5 (F(1,19)=38.48, p=0.000006, partial η^2^=0.67).


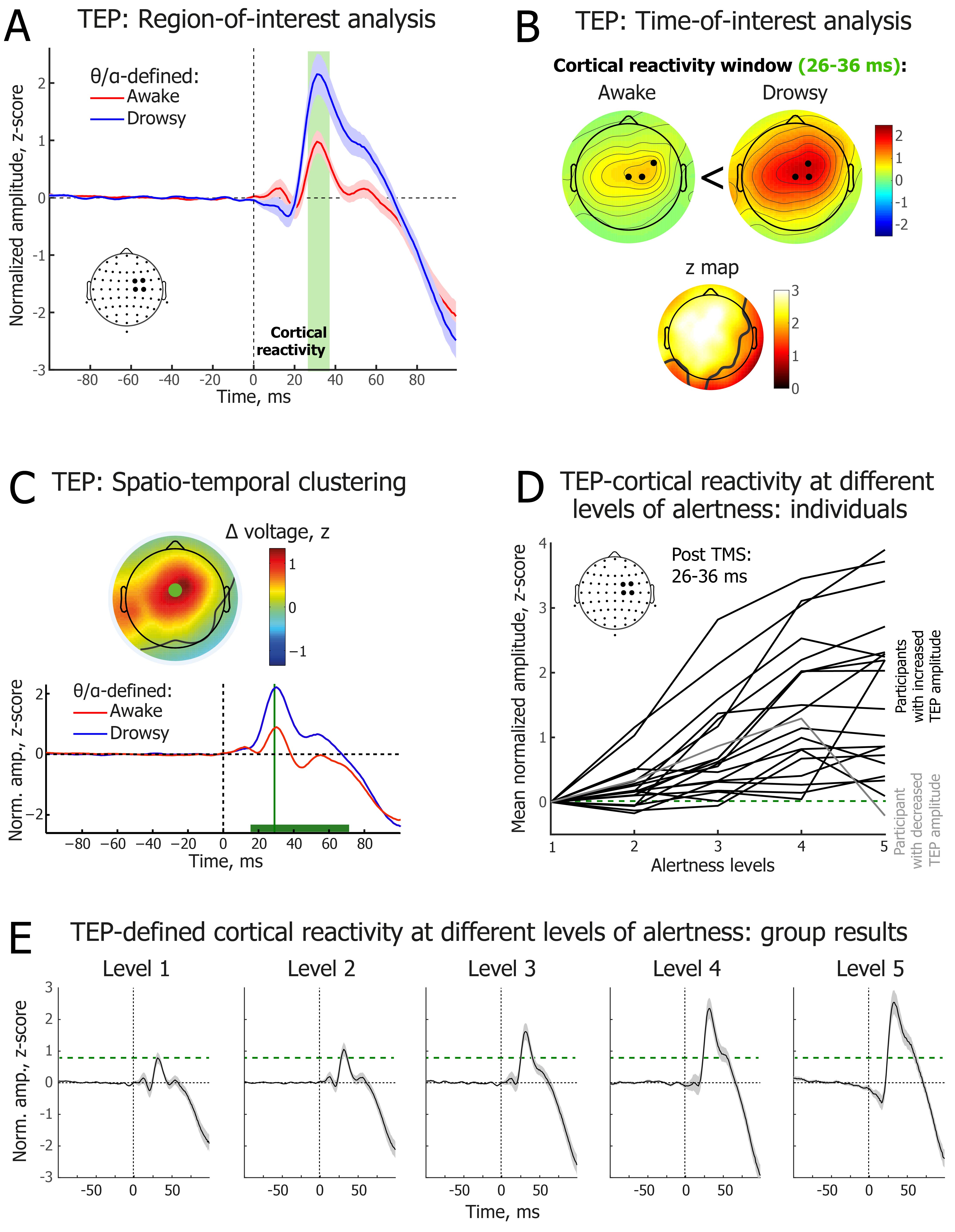


**Figure A.10 | Transcranial magnetic stimulation-triggered cortical reactivity potentials (TEPs) across different levels of alertness: Control analysis with auditory independent components in the z-score domain.** (A) Time course of electroencephalography (EEG) potentials averaged over 4 EEG electrodes beneath the TMS coil in the θ/α-defined awake (red) and drowsy (blue) trials. Green shaded area highlights the cortical reactivity time window (26-36 ms). Only behaviourally responsive trials are included in the analysis shown in this and other subplots. 0 ms corresponds to the time of the TMS pulse. Red and blue shading depicts standard error of the mean (SEM). There was a significant increase in TEP amplitude in the drowsy state relative to the awake state (t(19)=5.63, p=0.00002, d=0.94). (B) Topographical distribution of the early TEP mean peak at 26-36 ms post-TMS pulse in the θ/α-defined awake (upper left) and drowsy (upper right) states. Black dots indicate locations of three EEG electrodes with the maximal amplitude in the map. Non-parametric z map (below) reveals region significantly different between awake and drowsy states. (C) 0-100 ms data-driven spatio-temporal clustering of EEG potentials post-TMS pulse between θ/α-defined awake (red) and drowsy (blue) states. TEP amplitude was significantly higher in drowsy trials in a 15-71 ms time window (cluster peak: 29 ms, t=10578.11, p=0.002). The green horizontal line depicts the time window of significant difference. The electrode with the largest difference between awake and drowsy states is marked as a green dot in the topographic voltage map, and its waveforms are plotted below. The black contours within the map show the electrodes with statistically significant differences (cluster). The topographic voltage map is at the peak difference between awake and drowsy states. (D) Individual-level dynamics of TEP cortical reactivity peak amplitude across Alertness Levels 1-5 (TEP amplitude averaged over 26-36 ms across 4 electrodes beneath the TMS coil). Amplitude is shown relative to Alertness Level 1 (green dashed line). Black lines represent participants with higher TEP amplitude at Alertness Level 5 relative to Alertness Level 1 (N=19); grey lines represent participants with lower TEP amplitude at Alertness Level 5 relative to Alertness Level 1 (N=1). (E) Group-level dynamics of TEP waveforms across Alertness Levels 1-5 (TEPs averaged over 4 electrodes beneath the TMS coil). Horizontal green dashed line delineates TEP cortical reactivity peak at 31 ms post-TMS at Alertness Level 1. A linear trend of increasing TEP amplitude was observed across Alertness Levels 1-5 (F(1,19)=42.36, p=0.000003, partial η^2^=0.69).


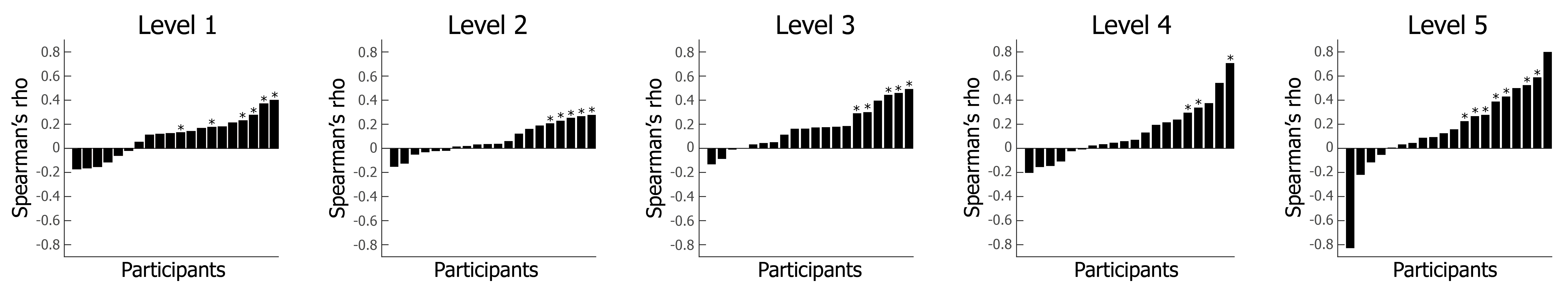


**Figure A.11 | Association between MEP and TEP amplitudes at different levels of alertness.** Bars represent intra-individual Spearman’s rank order correlation coefficients for the 20 participants, sorted from the least to the most positive coefficients. Asterisks above bars indicate individual datasets with a significant correlation between MEP and TEP amplitudes (p<0.05).

**Appendix B – Supplementary table**

**Table A.1. Numbers of trials used for the TEP amplitude analysis per TMS intensity condition (1-9) per state of alertness (θ/α-defined awake and drowsy states) per participant (1-20)**

| **TMS cond.** | | | **1** | |  | **2** | |  | **3** | |  | **4** | |  | **5** | |  | **6** | |  | **7** | |  | **8** | |  | **9** | | |
| --- | --- | --- | --- | --- | --- | --- | --- | --- | --- | --- | --- | --- | --- | --- | --- | --- | --- | --- | --- | --- | --- | --- | --- | --- | --- | --- | --- | --- | --- |
| **Alertness** | | | **A** | **D** |  | **A** | **D** |  | **A** | **D** |  | **A** | **D** |  | **A** | **D** |  | **A** | **D** |  | **A** | **D** |  | **A** | **D** |  | **A** | **D** | |
|  | | | | | | | | | | | | | | | | | | | | | | | | | | | | |  |
| **Participants** | **1** |  | 23 | 16 |  | 13 | 14 |  | 10 | 15 |  | 26 | 25 |  | 45 | 44 |  | 28 | 33 |  | 12 | 16 |  | 16 | 7 |  | 15 | 18 | |
|  | **2** |  | 17 | 15 |  | 20 | 13 |  | 9 | 19 |  | 34 | 31 |  | 45 | 46 |  | 41 | 28 |  | 13 | 22 |  | 14 | 23 |  | 19 | 15 | |
|  | **3** |  | 13 | 6 |  | 15 | 13 |  | 12 | 12 |  | 25 | 23 |  | 34 | 44 |  | 28 | 23 |  | 12 | 17 |  | 16 | 15 |  | 12 | 14 | |
|  | **4** |  | 9 | 9 |  | 8 | 6 |  | 10 | 7 |  | 16 | 16 |  | 23 | 28 |  | 28 | 22 |  | 8 | 12 |  | 10 | 10 |  | 11 | 13 | |
|  | **5** |  | 12 | 12 |  | 6 | 8 |  | 13 | 12 |  | 16 | 25 |  | 40 | 29 |  | 26 | 26 |  | 12 | 11 |  | 12 | 9 |  | 8 | 13 | |
|  | **6** |  | 13 | 5 |  | 11 | 9 |  | 9 | 9 |  | 23 | 19 |  | 40 | 28 |  | 21 | 28 |  | 11 | 14 |  | 7 | 14 |  | 10 | 19 | |
|  | **7** |  | 10 | 14 |  | 18 | 13 |  | 16 | 9 |  | 29 | 32 |  | 36 | 37 |  | 25 | 44 |  | 17 | 9 |  | 14 | 20 |  | 18 | 5 | |
|  | **8** |  | 14 | 18 |  | 17 | 14 |  | 13 | 18 |  | 33 | 31 |  | 50 | 44 |  | 36 | 28 |  | 17 | 16 |  | 11 | 20 |  | 16 | 18 | |
|  | **9** |  | 13 | 12 |  | 19 | 14 |  | 12 | 17 |  | 30 | 39 |  | 49 | 35 |  | 27 | 24 |  | 12 | 19 |  | 20 | 10 |  | 8 | 20 | |
|  | **10** |  | 13 | 12 |  | 23 | 11 |  | 15 | 19 |  | 30 | 25 |  | 48 | 38 |  | 24 | 22 |  | 5 | 22 |  | 13 | 12 |  | 9 | 19 | |
|  | **11** |  | 16 | 14 |  | 19 | 10 |  | 10 | 15 |  | 25 | 26 |  | 30 | 39 |  | 24 | 26 |  | 17 | 14 |  | 14 | 11 |  | 15 | 15 | |
|  | **12** |  | 14 | 11 |  | 16 | 17 |  | 17 | 12 |  | 26 | 32 |  | 51 | 45 |  | 29 | 32 |  | 16 | 13 |  | 8 | 14 |  | 11 | 12 | |
|  | **13** |  | 17 | 9 |  | 13 | 14 |  | 13 | 14 |  | 31 | 28 |  | 36 | 54 |  | 38 | 26 |  | 15 | 19 |  | 15 | 17 |  | 18 | 15 | |
|  | **14** |  | 11 | 17 |  | 10 | 13 |  | 21 | 6 |  | 27 | 36 |  | 41 | 30 |  | 21 | 31 |  | 15 | 14 |  | 16 | 13 |  | 12 | 14 | |
|  | **15** |  | 9 | 23 |  | 19 | 13 |  | 16 | 16 |  | 28 | 34 |  | 48 | 45 |  | 36 | 33 |  | 18 | 9 |  | 15 | 13 |  | 16 | 19 | |
|  | **16** |  | 17 | 15 |  | 13 | 14 |  | 18 | 14 |  | 25 | 24 |  | 35 | 39 |  | 28 | 28 |  | 17 | 10 |  | 12 | 19 |  | 10 | 12 | |
|  | **17** |  | 15 | 10 |  | 10 | 12 |  | 18 | 14 |  | 30 | 32 |  | 39 | 36 |  | 28 | 27 |  | 19 | 14 |  | 11 | 15 |  | 10 | 20 | |
|  | **18** |  | 15 | 18 |  | 21 | 15 |  | 17 | 16 |  | 35 | 27 |  | 49 | 47 |  | 22 | 37 |  | 18 | 14 |  | 13 | 24 |  | 21 | 13 | |
|  | **19** |  | 17 | 10 |  | 16 | 9 |  | 15 | 15 |  | 30 | 19 |  | 42 | 40 |  | 25 | 29 |  | 8 | 18 |  | 12 | 18 |  | 10 | 17 | |
|  | **20** |  | 15 | 10 |  | 11 | 15 |  | 8 | 17 |  | 26 | 21 |  | 35 | 50 |  | 34 | 24 |  | 19 | 8 |  | 15 | 14 |  | 13 | 17 | |
|  | | | | | | | | | | | | | | | | | | | | | | | | | | | | |  |
| **t** | |  | 1.09 | |  | 2.57 | |  | -0.15 | |  | 0.00 | |  | 0.45 | |  | -0.05 | |  | -0.31 | |  | -1.28 | |  | -1.69 | | |
| **p** | |  | 0.29 | |  | 0.02 | |  | 0.88 | |  | 1.00 | |  | 0.66 | |  | 0.96 | |  | 0.76 | |  | 0.21 | |  | 0.11 | | |

**Note:** A=awake state; D=drowsy state; t=paired samples t test statistic; p=significance value.

**Table A.2. MEP peak-to-peak amplitude: linear regression models with TMS intensity and EEG θ/α power ratio as predictors**

| **ID** |  | **Model 1: TMS intensity** | | |  | **Model 2: EEG θ/α** | | |  | **Model 3: TMS intensity, EEG θ/α** | | |
| --- | --- | --- | --- | --- | --- | --- | --- | --- | --- | --- | --- | --- |
|  |  | **R^2^** | **F** | **p** |  | **R^2^** | **F** | **p** |  | **R^2^** | **F change^a^** | **F change p** |
|  | | | | | | | | | | | | |
| **1** |  | 0.122 | 52.031 | 3E-12 |  | 0.006 | 2.420 | 0.121 |  | 0.127 | 1.924 | 0.166 |
| **2** |  | 0.229 | 125.272 | 1E-25 |  | 0.010 | 4.354 | 0.038 |  | 0.230 | 0.729 | 0.394 |
| **3** |  | 0.220 | 93.857 | 1E-19 |  | 0.038 | 13.045 | 0.0004 |  | 0.241 | 9.208 | 0.003 |
| **4** |  | 0.148 | 42.383 | 4E-10 |  | 0.001 | 0.131 | 0.717 |  | 0.148 | 0.048 | 0.827 |
| **5** |  | 0.148 | 50.204 | 1E-11 |  | 0 | 0.028 | 0.868 |  | 0.149 | 0.142 | 0.707 |
| **6** |  | 0.304 | 125.792 | 2E-24 |  | 0 | 0.020 | 0.888 |  | 0.316 | 5.030 | 0.026 |
| **7** |  | 0.345 | 192.036 | 2E-35 |  | 0.005 | 1.810 | 0.179 |  | 0.348 | 1.607 | 0.206 |
| **8** |  | 0.252 | 138.883 | 8E-28 |  | 0.029 | 12.458 | 0.0005 |  | 0.271 | 10.372 | 0.001 |
| **9** |  | 0.329 | 184.940 | 1E-34 |  | 0.055 | 22.198 | 0.000003 |  | 0.371 | 25.205 | 8E-7 |
| **10** |  | 0.176 | 76.697 | 8E-17 |  | 0.001 | 0.327 | 0.568 |  | 0.183 | 2.991 | 0.085 |
| **11** |  | 0.309 | 150.952 | 6E-29 |  | 0.001 | 0.420 | 0.518 |  | 0.311 | 1.077 | 0.300 |
| **12** |  | 0.263 | 133.786 | 1E-26 |  | 0.009 | 3.392 | 0.066 |  | 0.278 | 7.487 | 0.007 |
| **13** |  | 0.093 | 40.122 | 7E-10 |  | 0.031 | 12.529 | 0.0005 |  | 0.126 | 14.709 | 0.0001 |
| **14** |  | 0.213 | 93.539 | 1E-19 |  | 0.004 | 1.285 | 0.258 |  | 0.217 | 1.989 | 0.159 |
| **15** |  | 0.423 | 299.210 | 1E-50 |  | 0.005 | 1.972 | 0.161 |  | 0.424 | 0.498 | 0.481 |
| **16** |  | 0.005 | 1.735 | 0.189 |  | 0 | 0.077 | 0.781 |  | 0.005 | 0.033 | 0.857 |
| **17** |  | 0.276 | 136.689 | 6E-27 |  | 0.106 | 42.541 | 2E-10 |  | 0.362 | 47.735 | 2E-11 |
| **18** |  | 0.397 | 276.713 | 4E-48 |  | 0 | 0 | 0.999 |  | 0.399 | 1.393 | 0.239 |
| **19** |  | 0.203 | 88.640 | 7E-19 |  | 0.059 | 21.998 | 0.000004 |  | 0.228 | 11.438 | 0.001 |
| **20** |  | 0.245 | 113.548 | 4E-23 |  | 0.009 | 3.163 | 0.076 |  | 0.257 | 5.748 | 0.017 |
|  | | | | | | | | | | | | |
| **Mean:** |  | 0.235 | 120.852 | p<0.05 in 19/20 |  | 0.018 | 7.208 | p<0.05 in 7/20 |  | 0.250 | 7.468 | p<0.05 in 9/20 |
| **SD:** |  | 0.103 | 75.421 |  |  | 0.028 | 10.966 |  |  | 0.107 | 11.414 |  |
| **Min:** |  | 0.005 | 1.735 | 1E-50 |  | 0 | 0 | 2E-10 |  | 0.005 | 0.033 | 2E-11 |
| **Max:** |  | 0.423 | 299.210 | 0.189 |  | 0.106 | 42.541 | 0.999 |  | 0.424 | 47.735 | 0.857 |

**Note:** ^a^ Model 3 was compared with Model 1.

**Table A.3. TEP mean amplitude: linear regression models with TMS intensity and EEG θ/α power ratio as predictors**

| **ID** |  | **Model 1: TMS intensity** | | |  | **Model 2: EEG θ/α** | | |  | **Model 3: TMS intensity, EEG θ/α** | | |
| --- | --- | --- | --- | --- | --- | --- | --- | --- | --- | --- | --- | --- |
|  |  | **R^2^** | **F** | **p** |  | **R^2^** | **F** | **p** |  | **R^2^** | **F change^a^** | **F change p** |
|  | | | | | | | | | | | | |
| **1** |  | 0.008 | 2.972 | 0.086 |  | 0.018 | 6.778 | 0.010 |  | 0.027 | 7.175 | 0.008 |
| **2** |  | 0.119 | 57.071 | 3E-13 |  | 0.137 | 67.115 | 3E-15 |  | 0.226 | 58.018 | 2E-13 |
| **3** |  | 0.005 | 1.660 | 0.199 |  | 0.018 | 6.187 | 0.013 |  | 0.026 | 7.003 | 0.009 |
| **4** |  | 0.003 | 0.661 | 0.417 |  | 0.002 | 0.395 | 0.530 |  | 0.004 | 0.422 | 0.516 |
| **5** |  | 0.049 | 14.941 | 0.0001 |  | 0.133 | 44.217 | 1E-10 |  | 0.187 | 48.632 | 2E-11 |
| **6** |  | 0.002 | 0.591 | 0.443 |  | 0.018 | 5.381 | 0.021 |  | 0.023 | 6.262 | 0.013 |
| **7** |  | 0.006 | 2.264 | 0.133 |  | 0.102 | 41.190 | 4E-10 |  | 0.107 | 40.758 | 5E-10 |
| **8** |  | 0.008 | 3.207 | 0.074 |  | 0.033 | 14.237 | 0.0002 |  | 0.039 | 13.386 | 0.0003 |
| **9** |  | 0.239 | 118.543 | 3E-24 |  | 0.085 | 34.969 | 7E-9 |  | 0.309 | 38.382 | 2E-9 |
| **10** |  | 0.054 | 20.508 | 0.000008 |  | 0.086 | 33.616 | 1E-8 |  | 0.125 | 28.932 | 1E-7 |
| **11** |  | 0.044 | 15.741 | 0.00009 |  | 0.008 | 2.843 | 0.093 |  | 0.052 | 2.685 | 0.102 |
| **12** |  | 0.013 | 4.748 | 0.030 |  | 0 | 0.140 | 0.709 |  | 0.013 | 0.072 | 0.789 |
| **13** |  | 0.002 | 0.656 | 0.418 |  | 0.087 | 37.154 | 3E-9 |  | 0.088 | 36.943 | 3E-9 |
| **14** |  | 0.067 | 25.040 | 9E-7 |  | 0.119 | 46.710 | 4E-11 |  | 0.184 | 49.263 | 1E-11 |
| **15** |  | 0.036 | 15.339 | 0.0001 |  | 0.029 | 12.348 | 0.0005 |  | 0.070 | 14.874 | 0.0001 |
| **16** |  | 0.050 | 18.468 | 0.00002 |  | 0.002 | 0.788 | 0.375 |  | 0.051 | 0.348 | 0.555 |
| **17** |  | 0.231 | 107.593 | 3E-22 |  | 0.095 | 37.523 | 2E-9 |  | 0.304 | 39.627 | 9E-10 |
| **18** |  | 0.012 | 5.301 | 0.022 |  | 0.002 | 0.845 | 0.358 |  | 0.014 | 0.582 | 0.446 |
| **19** |  | 0.014 | 5.004 | 0.026 |  | 0.011 | 3.860 | 0.050 |  | 0.021 | 2.456 | 0.118 |
| **20** |  | 0.022 | 8.030 | 0.005 |  | 0.012 | 4.086 | 0.044 |  | 0.035 | 4.561 | 0.033 |
|  | | | | | | | | | | | | |
| **Mean:** |  | 0.049 | 21.417 | p<0.05 in 13/20 |  | 0.050 | 20.019 | p<0.05 in 14/20 |  | 0.095 | 20.019 | p<0.05 in 14/20 |
| **SD:** |  | 0.070 | 34.009 |  |  | 0.049 | 20.486 |  |  | 0.096 | 20.032 |  |
| **Min:** |  | 0.002 | 0.591 | 3E-24 |  | 0 | 0.140 | 3E-15 |  | 0.004 | 0.072 | 2E-13 |
| **Max:** |  | 0.239 | 118.543 | 0.443 |  | 0.137 | 67.115 | 0.709 |  | 0.309 | 58.018 | 0.789 |

**Note:** ^a^ Model 3 was compared with Model 1.
